# Integrated Quantitative Targeted Lipidomics and Proteomics Reveal Unique Fingerprints of Multiple Metabolic Conditions

**DOI:** 10.3390/biom12101439

**Published:** 2022-10-08

**Authors:** Anna A. Ivanova, Jon C. Rees, Bryan A. Parks, Michael Andrews, Michael Gardner, Eunice Grigorutsa, Zsuzsanna Kuklenyik, James L. Pirkle, John R. Barr

**Affiliations:** Division of Laboratory Sciences, National Center for Environmental Health, Centers for Disease Control and Prevention, Atlanta, GA 30341, USA

**Keywords:** dyslipidemias, lipidomics, proteomics, network analysis, artificial neural network classification

## Abstract

Aberrations in lipid and lipoprotein metabolic pathways can lead to numerous diseases, including cardiovascular disease, diabetes, neurological disorders, and cancer. The integration of quantitative lipid and lipoprotein profiling of human plasma may provide a powerful approach to inform early disease diagnosis and prevention. In this study, we leveraged data-driven quantitative targeted lipidomics and proteomics to identify specific molecular changes associated with different metabolic risk categories, including hyperlipidemic, hypercholesterolemic, hypertriglyceridemic, hyperglycemic, and normolipidemic conditions. Based on the quantitative characterization of serum samples from 146 individuals, we have determined individual lipid species and proteins that were significantly up- or down-regulated relative to the normolipidemic group. Then, we established protein–lipid topological networks for each metabolic category and linked dysregulated proteins and lipids with defined metabolic pathways. To evaluate the differentiating power of integrated lipidomics and proteomics data, we have built an artificial neural network model that simultaneously and accurately categorized the samples from each metabolic risk category based on the determined lipidomics and proteomics profiles. Together, our findings provide new insights into molecular changes associated with metabolic risk conditions, suggest new condition-specific associations between apolipoproteins and lipids, and may inform new biomarker discovery in lipid metabolism-associated disorders.

## 1. Introduction

Lipid metabolism plays a central role in maintaining the normal homeostasis of the human body. Aberration of lipid metabolism is a trigger for chronic diseases, including diabetes, neurological disorders, cancer, and cardiovascular disease (CVD) [1,2,3]. Traditionally, screening among asymptomatic individuals for lipid disorders rests on consideration of age, gender, blood pressure, smoking status, and testing for cholesterol, triglycerides, and glucose levels in plasma. Cholesterol tests measure both endogenous free cholesterol (FC) and hydrolyzed cholesteryl esters (CEs). The cholesterol content of the density or size fractions of lipid-carrying lipoprotein particles in plasma allows estimation of the particle number of high-density (HDL), low-density (LDL), very low-density (VLDL) lipoproteins, and chylomicrons [4,5]. In the case of CVD especially, these traditional lipid profile measures leave many individuals undiagnosed [6]. The prevention and treatment of dyslipidemias require the development of alternative diagnostic tools that allow for the assessment of the lipid and protein constituents of lipoproteins that are more directly related to the underlying unique metabolic irregularities of individuals [7,8,9,10,11].

The main lipid constituents of lipoproteins are CEs and triacylglycerols (TAGs) contained within their core, amphipathic phospholipids (PLs) and ceramides (CERs) on their surface, and FC distributed between the core and the surface. The main PLs are phosphatidylcholines (PCs), phosphatidylethanolamines (PEs), sphingomyelins (SMs), and lyso-derivatives (LPCs and LPEs). These lipid classes have high structural diversity. The cholesteryl, glycero-phosphatidyl, glyceryl, and sphingosine backbones carry fatty acyl moieties (FAs) that are linked through ester, ether, or amide bonds. FAs differ by carbon chain length (usually between 12 and 24) and their number of double bonds (depending on the chain length between 0 and 6). In addition, lipid molecules may differ by the position of FA groups on the backbone, carbohydrate modifications of the backbone, and the position of double bonds on the FA chain.

With the polar PL head groups exposed to the aqueous plasma environment, the surface of the lipoproteins incorporates numerous types of proteins [12,13,14]. An important class of lipoprotein binding proteins is apolipoproteins (apos), with amphipathic alpha-helical domains that have a unique affinity to phospholipid monolayers. Apos are essential for the biogenesis, structural integrity, and function of lipoprotein particles [15,16]. Structurally, one of the most essential apo is apoA1 for HDL and apoB-100 for LDL and VLDL formation [15,17]. Chylomicrons contain a truncated form of apoB (apoB-48) [18], and on the more atherogenic Lp(a) particles, apoB is extended through the S-S bond by apo(a) [19]. Apos that interact with apoA1 and apoB containing lipoproteins, called exchangeable apos and have well-characterized roles as cofactors and inhibitors in lipoprotein remodeling processes include A2 [20], A4 [21], C1 [22], C2 [23], C3 [23], and E [24]. Other exchangeable proteins are known as non-polar molecule carriers (apoD and apoM), lipid transfer proteins (CETP and PLTP), lipase enzymes (LCAT), or inflammation-related proteins (AACT, HP, PON1, SAA1, SAA4, and TF) [12,13].

The lipid and protein constituents together affect the structural integrity as well as the metabolic fate and circulating plasma concentration of lipoproteins. The competitive binding of exchangeable apos is modulated by the fluidity of the phospholipid monolayer that is determined by species composition, studied mostly by using model membranes and purified or artificial lipoproteins [25,26,27,28,29,30,31]. Advances in tandem mass spectrometry detection (MS/MS) techniques and the availability of stable isotope-labeled internal standards [32,33,34,35] enable a steadily increasing number of both lipid species and proteins that can be quantified from one sample [36,37,38,39]. The integration of these quantitative lipidomics and proteomics techniques brings promising opportunities not only in basic research but also in epidemiology and diagnostics [32,40,41,42].

In this study, we leveraged a multi-omics approach to determine lipidomic and proteomic profile changes associated with different metabolic risk categories: hypercholesterolemic (HC), hypertriglyceridemic (HT), hyperlipidemic (HL), and hyperglycemic (HG), relative to a normolipidemic (NL) control group. By discriminant analysis, we have found unique concentration changes in both proteins and lipid species in each of the risk categories. Based on the concentration correlations between lipid species and proteins we constructed protein–lipid connectivity networks that provide new insights into lipid and protein constituents of lipoproteins unique to the individual metabolic categories. In support of our approach, the most significantly different lipid species and proteins and their concentration correlation patterns were consistent with known pathways of lipid synthesis and extracellular lipoprotein remodeling. Furthermore, using the machine learning artificial neural network (ANN) approach, known as “deep learning”, we have identified a set of lipids and proteins that can accurately distinguish among all four pathological conditions and controls. Together, our data provide new insights into molecular profiles of different metabolic conditions and demonstrate the potential of integrated multi-omics to improve the characterization and differentiation of metabolic disorders.

## 2. Materials and Methods

### 2.1. Chemicals and Reagents

HPLC grade methanol (MeOH), dichloromethane (DCM), 1-propanol, 2-propanol, sodium bicarbonate, sodium chloride, isopropanol, hexanes, ethanol, isopropanol, and water were purchased from Fisher Scientific (Waltham, MA, USA). Ammonium acetate (NH_4_AcO) was obtained from Millipore Sigma (St. Louis, MO, USA). Labeled d7-cholesterol was purchased from Sigma-Aldrich (St. Louis, MO, USA). Cholesteryl-d7-palmitate was purchased from Avanti Polar Lipids, (Alabaster, AL, USA). Labeled d98-tripalmitin was purchased from CDN Isotopes, (Pointe-Claire, QC, Canada). NIST standard reference materials were purchased from the National Institute of Standards and Technology (Gaithersburg, MD, USA). The internal standards kit for the Lipidyzer platform was purchased from AB SCIEX (Framingham, MA, USA). Labeled internal standard peptides for proteomics analysis were purchased from Biosynth (Gardner, MA, USA). Human plasma samples pooled for quality control (QC) were purchased from BioIVT, Inc. (Westbury, NY, USA).

### 2.2. Study Population and Samples

Serum samples from 146 specimens were purchased from BioIVT, Inc. (Westbury, NY, USA). The study population included 81 males (55%) and 65 females (45%), with a mean age of 60.6 ± 17.2 years. All samples were collected from individuals fasting for more than 8 h. Based on clinically measured levels of total cholesterol (Total-C), total triglycerides (Total-TAG), and glucose, the samples were ordered from five categories. The mean levels in the sample groups were as follows: hypercholesteremia, HC (*n* = 36, 274 (245–310) mg/dL Total-C and 93 (50–125) mg/dL Total-TAG); hypertriglyceridemia, HT (*n* = 32, 188 (136–236) mg/dL Total-C and 268 (161–380) mg/dL Total-TAG); hyperlipidemia, HL (*n* = 28, 279 (232–355) mg/dL Total-C and 299 (155–573) mg/dL Total-TAG); hyperglycemia HG (*n* = 29, >180 mg/dL glucose, 122 (82–164) mg/dL Total-C and 146 (26–459) mg/dL Total-TAG); and a normolipidemic, NL group (*n* = 21, Total-C < 200 mg/dL, Total-TAG < 150 mg/dL, glucose < 180 mg/dL). Of note, these Total-C (FC + CE) and Total-TAG measurements were obtained with our in-house developed LC-MS/MS method described in detail in ref. [43]. Briefly, the sample extraction was conducted in 96-well plates. Each sample was extracted in a single well, “in one-pot”, without the need for manual liquid phase separation or sample transfer before LC-MS/MS analysis. For the simultaneous analysis of FC, CE, and TAG species, UHPLC separation and in-source collision-induced dissociation (CID) coupled MS/MS method was used. Aliquots of 50 μL of 1:100 dilute serum samples in 10 mm sodium bicarbonate and 75 mm sodium chloride pH = 7.4 buffer were placed on a 96-well plate. Cholesteryl palmitate was used as the external calibrator for the CE lipid class and the mixture of triolein, tripalmitin, and trilinolein in a ratio of 514:313:173, reflective of the typical ratio in humans, was used as an external calibrator for the TAG lipid class. QCs prepared from NIST SRM 1951c were analyzed with each plate. The internal standard (IS) spiking mix of stable isotope labeled analogs were prepared in ethanol, containing 0.033 mg/dL d7-cholesterol (IS for FC), 0.098 mg/dL cholesteryl-d7-palmitate (IS for CE), and 0.125 mg/dL d98-tripalmitin (IS for TAG). The UHPLC system Agilent 1290 (Agilent Technologies, Santa Clara, CA, USA) coupled to a hybrid triple quadrupole/linear ion trap Sciex 4000 QTrap (Sciex, Framingham, MA, USA) was used. The column was a Kinetex HILIC 1.7 μm, 2.1 × 50 mm (Phenomenex, Torrance, CA, USA). Mobile phase A was hexanes with 0.05% isopropanol. Mobile phase B was hexanes with 5% ethanol and 0.05% isopropanol. Class-specific fragments were generated in-source for CE and TAG prior to MS/MS. The multiple reaction monitoring (MRM) method in positive ion mode was used for data acquisition. Collected raw data were processed with Multiquant software.

### 2.3. Ethics Approval and Consent to Participate

All samples were de-identified prior to shipment such that no personal identification was associated with any sample. The project was approved as research not involving identifiable human subjects under the U.S. Health and Human Services Department Policy for Protection of Human Research Subjects codified of Federal Regulations at 45 CFR part 46.

### 2.4. Sample Preparation and Targeted Lipidomics Analysis

Lipids were extracted using a modified Bligh and Dyer extraction protocol [44]. Briefly, 2 mL methanol, 1 mL dichloromethane (DCM), and 1 mL water were added to 25 µL serum samples containing one or two internal standards for each lipid class. The list of deuterium-labeled internal standards spiked into all quality controls, and unknowns can be found in Supplementary Table S1. The generated monophase mixture was incubated at room temperature (20 ± 2 °C) for 30 min followed by the addition of 1 mL water and 0.9 mL DCM, gentle mixing, and 10-min centrifugation at 1200 RPM to assist in phase separation. The lower layer containing DCM was transferred to a separate tube, and the lipid extraction was repeated a second time. All collected lower phases containing lipids were evaporated under nitrogen to dryness and reconstituted with 250 µL buffer containing 50:50 (*v*:*v*) DCM:MeOH and 10 mM NH_4_AcO.

In an earlier study using a similarly grouped sample set, we reported that HDL particles have lower SM/PL and higher PE/PL molar ratios than LDL and VLDL particles [45]. Within HDL, LDL, and VLDL fractions, we found higher SM/PL and lower PE/PL ratios in HC and NL than in HT and HL samples. Consequently, the direction of these trends was similarly observed in unfractionated samples as well. Therefore, in this study, we analyzed lipids and proteins without fractionation and assumed that main lipid composition differences between sample groups would similarly apply to HDL, LDL, and VLDL fractions as well.

The Lipidyzer platform (AB SCIEX, Framingham, MA, USA) was used to detect and quantify lipid concentrations in the serum extracts, as described in detail elsewhere [46]. A 50 µL aliquot of the extracts was injected into a constant 50 µL/min flow of 50:50 (*v*:*v*) DCM:MeOH and 10mM NH_4_AcO buffer and directly infused into the triple quadrupole SCIEX QTRAP 5500 mass spectrometer. The infusion was repeated using two different acquisition methods, both containing polarity switching in positive and negative modes. The first method used the SelexION Differential Mobility Spectrometry (DMS) to analyze PC, PE, SM, LPC, and LPE species. The second method was run without the DMS to select for and analyze TAG, DAG, CER, CE, and FFA species. Each method cycles through its respective list of MRM scans twenty times, and all quantitation was accomplished using the average signal of the twenty cycles for both native lipid species and internal standards to calculate response ratios. The response ratios were multiplied with the respective spiked internal standard concentrations to obtain species concentration. All species except TAGs were quantified based on a single unique MS/MS signal relative to the analogous MS/MS signal of a labeled internal standard specie (Supplementary Table S1). TAGs were monitored by 1–3 MRM transitions.

Lipid species had to have 10–20 %CVs and <30% missing values to be considered quantifiable. After applying these criteria, the list of species included 12 SMs, 9 LPCs, 4 LPEs, 22 CEs, 22 PCs, 20 PEs, 6 CERs, 4 HCERs, 23 FFAs, 17 DAGs, and 435 TAGs. The coefficient of variation (CV) for each lipid class was calculated using quality control (QC) samples (Supplementary Table S2).

Summing the molar concentration of lipid species by class yielded lipid class concentrations (Table 1). Some species had a low %Abundance of 0.01–3% within the lipid class. All quantified species had at least pmol/mL level of absolute concentrations and were quantifiable with 10–20 %CVs and <30% missing values, sufficient to find statistically significant changes relative to controls and find correlations with proteins within confidence intervals around mean concentrations of individual lipid species.

### 2.5. Categorization of Lipid Species by FA Carbon Chain Length and Saturation

We categorized the lipid species according to their number of double bonds on FA carbon chains (Supplementary Table S3). The FA groups were annotated as odd chain, saturated or mono-unsaturated (SFA/MUFA), double-unsaturated (DUFA), and poly-unsaturated (PUFA). Lipid species that had an odd number of total FA carbons, generally containing FA15:0 or FA17:0, were categorized as odd, regardless of the other FAs on the molecule. PCs, PEs, and DAGs were categorized based on the annotation of the FA with the greater number of double bonds. TAGs with an even number of total FA carbons were categorized based on the FA group with the greatest number of double bonds.

### 2.6. Sample Preparation and Targeted Proteomics Analysis

In this study, we conducted targeted proteomics analysis for a focused set of 20 apolipoproteins and proteins related to HDL, LDL, and VLDL remodeling. The proteomics data were acquired with a Perfinity IDP workstation (Shimadzu Scientific) using on-line protein digestion with an immobilized enzyme reactor (IMER) directly coupled to a HALO-C18 analytical column (Advanced Materials Technology, Wilmington, DE, USA). All samples were diluted 1:100 with buffer containing 10 mM NaHCO_3_ and 150 mM NaCl at pH 7.4. To a 100 μL aliquot from each diluted sample, a 50 μL of digest buffer containing 0.45% Zwittergent 3–12 was added. Then, samples were mixed on a shaker plate at 500 rpm for 5 min and placed directly into the autosampler at 8 °C for subsequent digestion and MRM analysis. A detailed protocol of the procedure can be found in Toth et al. [47]. Labeled peptide internal standards were co-injected with the sample onto the IMER (Supplementary Figure S7). Peptides were trapped on a C18 trapping column, which is subsequently switched in-line with an analytical column using the same stationary phase. Eluted peptides from the analytical column were directly analyzed by MRM on a QTRAP 6500 (AB SCIEX, Framingham, MA, USA). A dilution series of plasma-based calibrators and QCs that had been previously value-assigned for target proteins were analyzed with each sample plate, and the calibrators were used to generate calibration curves of peptide area ratio versus protein concentration. Targeted protein analysis method reproducibility was established using QC samples from pooled human plasma. Protein concentration CVs for the QC samples calculated for each protein are shown in Supplementary Table S2.

### 2.7. Data Processing and Statistical Analysis

Targeted lipidomics and proteomics mass spectrometry raw data processing was performed with the Lipidomics Workflow Manager (AB SCIEX, USA) and Multiquant (AB SCIEX, USA), respectively. The lipid species and protein concentration quantification were performed based on the signal intensity relative to the corresponding internal standard (Supplementary Table S4). Further data processing and formatting were performed using JMP Pro software (SAS Institute, Cary, NC, USA). Prior to statistical analysis, the lipid species and protein concentration data with more than 30% missing values and CV for QC > 30% were removed. After applying these criteria, missing values for remaining lipid species (574) and proteins (20) were imputed with one-half of the minimum value for each variable. The non-parametric Wilcoxon and Kruskal–Wallis tests implemented in JMP Pro were used for the evaluation of absolute plasma concentration differences. The false discovery rate (FDR)-adjusted q-values were calculated with the Benjamini–Hochberg procedure. Means comparison analysis of lipid and protein concentrations, cluster analysis, and lipid–protein correlation network analysis were conducted using custom R and Python scripts. Namely, the MetaboAnalyst and ggvenn R packages were used to build Venn diagrams, heatmaps, and conduct the clustering. The Pearson’s correlations, *p*-values, and q-values for the protein-correlation networks were calculated using the scipy.stats and statmodels Python libraries. The volcano plots were visualized using the matplotlib and seaborn Python libraries. Networks were visualized using the Cytoscape software [48]. Pathway enrichment analysis was performed based on the biological processes defined in Gene Ontology [49] and signaling and metabolic pathways defined in Reactome [50] databases using the Enrichr application programming interface (API) implemented in Python [51]. From now on, we will refer to the analysis of both the Reactome pathways and GO biological processes as “pathway analysis”. The *p*-value < 0.01 and q-value < 0.01 were used as the thresholds for statistical significance (unless noted). Predictor Screening procedures and the ANN modeling were conducted using the JMP Pro software.

## 3. Results

### 3.1. Comparative Assessment of Lipidomic Profiles across Different Metabolic Categories

**Figure 1:**
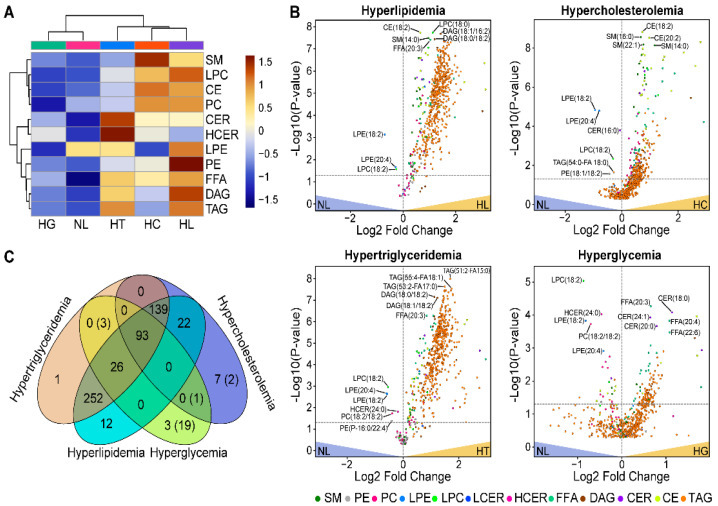
Dysregulated lipid classes and individual lipid species in metabolic groups. (**A**) Heat map comparison and clustering of lipid class concentrations for hyperlipidemia (HL), hypercholesterolemia (HC), hypertriglyceridemia (HT), hyperglycemia (HG), and normolipidemic (NL) samples. The lipid classes and sample groups are shown in rows and columns, respectively. The colors correspond to the Z-score normalized by the grand mean of each lipid class concentration. (**B**) Volcano plots show individual lipid species concentrations that were dysregulated relative to normal (NL) samples. The horizontal line on the plots shows a *p*-value ≤ 0.05. (**C**) The Venn diagram summarizes the number of lipid species significantly upregulated in different metabolic groups, the numbers in parentheses show the number for downregulated lipids.

First, we assessed the overall differences in the lipid class concentrations across different metabolic conditions. Using the Lipidyzer platform we have determined and quantified a total of 574 lipid species from 11 lipid classes (Table 1). The analysis of variance (ANOVA) showed that the average concentration of lipid classes varied significantly (*p*-value < 0.05) across the sample groups (Figure 1A, Table 1). Moreover, 8 classes, including CE, CER, DAG, FFA, LPC, PC, SM, and TAG, showed highly significant differentiation with *p*-values < 0.0001 (Table 1). Clustering analysis of concentrations by lipid classes revealed three lipid-class clusters: CER/HCER, TAG/PE/FFA/DAG/LPE, and CE/SM/PC/LPC (Figure 1A). The CER and HCER levels were highest in the HT and lowest in the NL group. The TAG containing cluster showed higher concentrations in the HT and HL than in the three other groups, while the concentrations of the CE containing cluster were higher for the HC and HL sample categories.

Then, we performed means comparison analysis by lipid species, comparing mean concentrations in NL samples to the HC, HT, HL, and HG samples (Figure 1B). Out of 574 species, we found that the concentrations of 267 lipids in the HC, 547 lipids in the HL, 517 lipids in the HT, and 148 lipids in the HG group were significantly different (*p*-value < 0.05) when compared to the NL samples (Figure 1B, Supplementary Table S3). The false discovery rate (FDR) for more than 95% of the significantly different lipid species was low, FDR < 1%, otherwise the FDR was 1–18%.

Most lipid species were up-regulated relative to NL, including 93 lipid species that were elevated in all four disorder groups (HT, HC, HL, and HG), 39 species in HT, HL, and HC, 26 species in HG along with HL and HT, 252 species in the groups with high triglyceride levels (HT and HL), and 22 in groups with high cholesterol (HC and HL) (Figure 1C, Supplementary Table S5). The number of species that uniquely increased in a single sample group was relatively small and worthy of mention, specifically: in the HC group PC(18:1/16:1), PC(18:1/18:1), SM(26:0), LCER(16:0), HCER(24:1), HCER(24:0), and HCER(22:0); in the HT group TAG(42:1-FA18:1); in the HL group PC(16:0/20:4), LPC(20:3), PE(P-16:0/18:1), PE(18:0/18:1), PE(18:0/18:2), PE(P-18:1/20:4), TAG(58:8-FA20:3), DAG(16:0/18:0), TAG(42:0-FA14:0), TAG(44:0-FA12:0), TAG(58:9-FA20:4), and PE(16:0/18:1); and in the HG group DAG(18:2/20:4), DAG(18:1/20:4), and DAG(16:0/20:4).

The concentration of species that were down-regulated compared to NL included LPE(20:4), LPE(18:2), LPC(18:2) in all sample groups; PC(18:2/18:2), PE(P-16:0/22:4), and HCER(24:0) in HT and HG; and TAG(54:0-FA18:0) in HC and HG; and 5 CEs, 7 PCs, 2 CERs, 2 SMs, 1 LPC, 2 TAGs in only HG (Figure 1B,C, Supplementary Tables S3 and S5).

**Figure 2:**
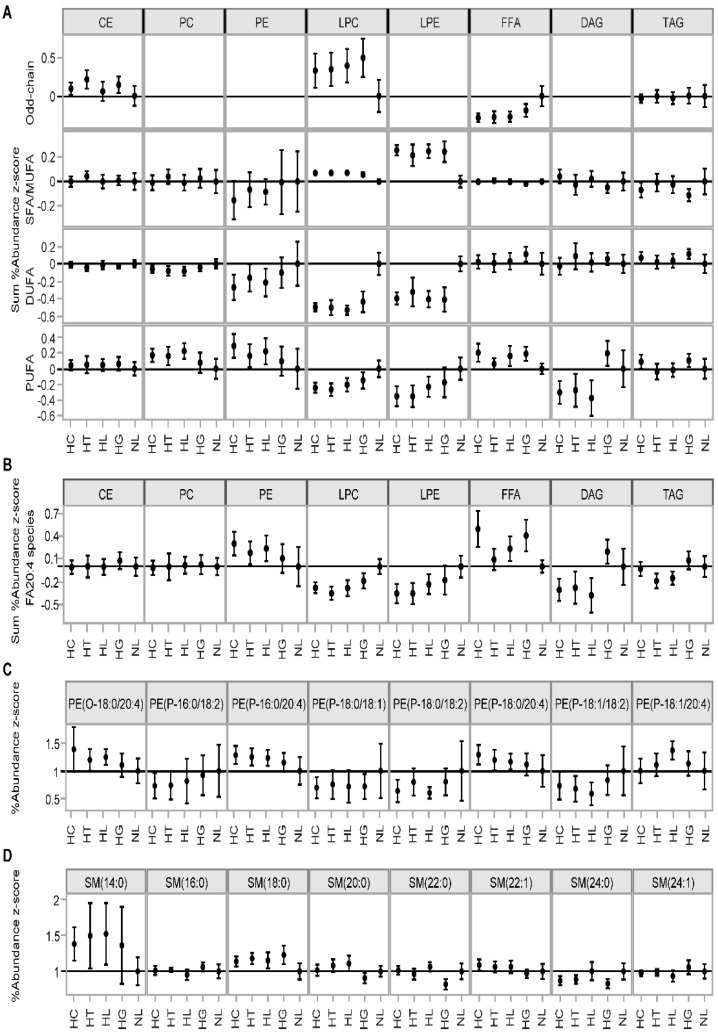
Comparison of %Abundances of lipid species by odd-chain, SFA/MUFA, DUFA, and PUFA containing sub-classes, normalized and compared to the NL group. Sum %Abundance by lipid classes (**A**), sum %Abundance of FA20:4 containing species (**B**), %Abundance of individual PE species (**C**) and SM species (**D**). Error bars represent confidence intervals.

Then, we sought to determine the common patterns in changes in lipid concentrations within the same lipid class. We assessed the lipid species abundance within lipid classes based on the FA group saturation. For each sample, we summed the lipid species concentrations within lipid classes by odd-chain FA, SFA/MUFA, DUFA, and PUFA containing sub-classes and divided by the total lipid class concentrations, obtaining %Abundance values (Figure 2A). Relative to NL, odd-chain FA containing CEs and LPCs were higher. LPCs and LPEs with SFA/MUFA were higher, and those with DUFA and PUFA were lower. Correspondingly, PC, PEs, and FFAs with PUFA were higher, but those with SFA/MUFA showed no significant difference. By closer examination of the individual species, we found that increase in FA(20:4) containing PEs corresponded with a lower abundance of FFA(20:4) (Figure 2B). This is consistent with the hydrolysis of PUFA-containing PCs and PEs, especially those with FA(20:4), to SFA/MUFA LPC/LPE and PUFA FFA products.

### 3.2. Comparative Assessment of Proteins

**Figure 3:**
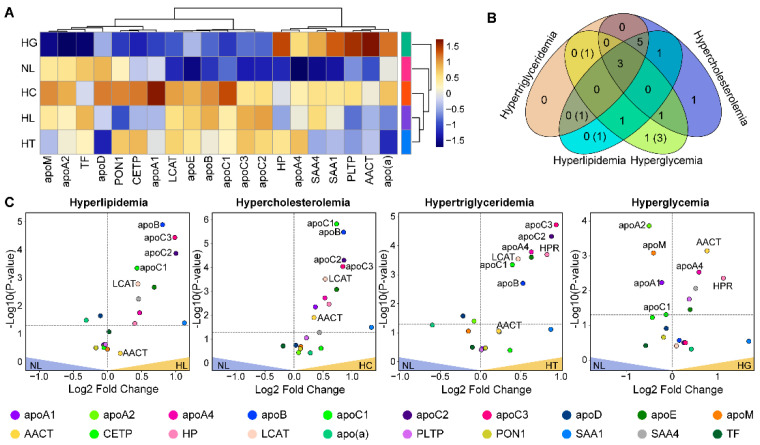
Dysregulated proteins in metabolic groups. (**A**) Heat map comparison and clustering of protein concentrations for hyperlipidemia (HL), hypercholesterolemia (HC), hypertriglyceridemia (HT), hyperglycemia (HG), and normolipidemic (NL) samples. The proteins and sample groups are shown in rows and columns, respectively. The colors correspond to the Z-score normalized by the grand mean of each protein concentration. (**B**) Volcano plots show individual protein concentrations that were dysregulated relative to normal (NL) samples. The horizontal line on the plots shows a *p*-value ≤ 0.05. (**C**) The Venn diagram summarizes the number of proteins significantly upregulated in different metabolic groups, the numbers in parentheses are for down-regulated proteins.

Then, we determined changes in protein concentrations associated with different metabolic conditions. As with lipid classes, we performed means comparison and cluster analysis of the protein concentrations (Figure 3A). HG samples appeared most similar to the NL samples, while HC, HL, and HT formed a separate cluster, and three major protein clusters were identified. The first cluster included apo(a), AACT, PLTP, SAA1, SAA4, HP, and apoA4, which showed higher levels of protein concentrations in HG samples as compared to other sample groups. The second cluster of apoA1, apoA2, CETP, PON1, apoD, apoM, and TF appeared up-regulated in HC samples. The third cluster included apoC1, apoB, LCAT, apoC2, apoC3, and apoE, which had higher concentrations in the HL, HC, and HT groups than in the HG and NL groups. Using one-way ANOVA analysis, we found that the average concentrations of 13 out of 20 proteins were significantly different across the sample groups (*p*-value < 0.05, FDR < 5%) (Table 2).

Compared to the NL samples, we found that 17 out of 20 proteins were significantly different (*p*-value < 0.05, FDR < 5%) in at least one of the other four sample categories (Supplementary Table S3). ApoE, apoA4, and HP were generally up-regulated relative to the NL group. Apos B, C1, C2, C3, and LCAT were up-regulated in HL, HC, and HT, but not in the HG group. SAA4 and AACT were elevated in HG and HL, and SAA1 appeared up-regulated in HL and HC groups. The HG group was significantly differentiated from the NL group by relative down-regulation of apos A1, A2, and C1. In addition, there was a unique up-regulation of PLTP and down-regulation of apoM in HG, and down-regulation of apoD in HT samples (Figure 3B,C).

### 3.3. Lipid–Protein Correlation Analysis

The correlations between lipid and protein concentrations within the same sample group may indicate functional or physical lipid–protein interaction. To determine such interactions, we closely examined the concentration correlations between proteins and lipids by sample group. We designated Pearson correlation coefficients of r > |0.3| and *p*-value < 0.05 as significant, and r > |0.5| and *p*-value < 0.002 as strong correlations (Supplementary Table S6). The strength of significant correlations between proteins and lipids did not correspond with their up- or down-regulation relative to the NL group (Supplementary Figure S1). Furthermore, the strengths of correlations did not follow ranks by %Abundance in the lipid classes (Supplementary Figure S2). Instead, the numbers and strengths of the correlations showed recognizable differences among species categories (odd-chain FA, SFA/MUFA, DUFA, and PUFA) for each lipid class and protein (Supplementary Figure S3). Therefore, we concluded that the strength of the lipid–protein correlation was not the result of simple coincidental associations and may indicate the participation of correlating lipids and proteins in the same lipoproteins remodeling processes.

We found that, in the NL group, apoB and apoC2 correlated strongest with odd-chain FA and PUFA CEs, while apoC3 correlated with SFA/MUFA and DUFA CEs, PCs, and TAGs (Supplementary Figure S3). A unique feature of the HC group was the negative correlation of apoA1 and apoA2 with TAGs containing mostly DUFA, PUFA, and odd-chain FAs, while much fewer SFA/MUFA. Moreover, there were positive correlations of apos B, C2, C3, and E with TAGs and PCs, with a higher preference for SFA/MUFA and DUFA TAGs and PCs. In the HT group, apos A1, B, and C1 showed positive correlations with TAGs containing mostly SFA/MUFA, while apos C2, C3, and E correlated with TAGs with a preference toward PUFA TAGs. The HL group was unique in terms of few correlations of apoA1 and apoA2 with lipid species, and instead, apoA4 was found to be correlating negatively with many DUFA and PUFA CEs, and positively with a high number of TAGs with SFA/MUFA, DUFA, and odd-chain FA. In the HG group, lipid species from all classes correlated strongly and positively with apos B, C1, C2, C3, and M, especially PUFA containing CEs, PCs, and DAGs, as well as odd-chain FA containing TAGs and FFAs.

### 3.4. Lipid–Protein Network Analysis by Sample Groups

**Figure 4:**
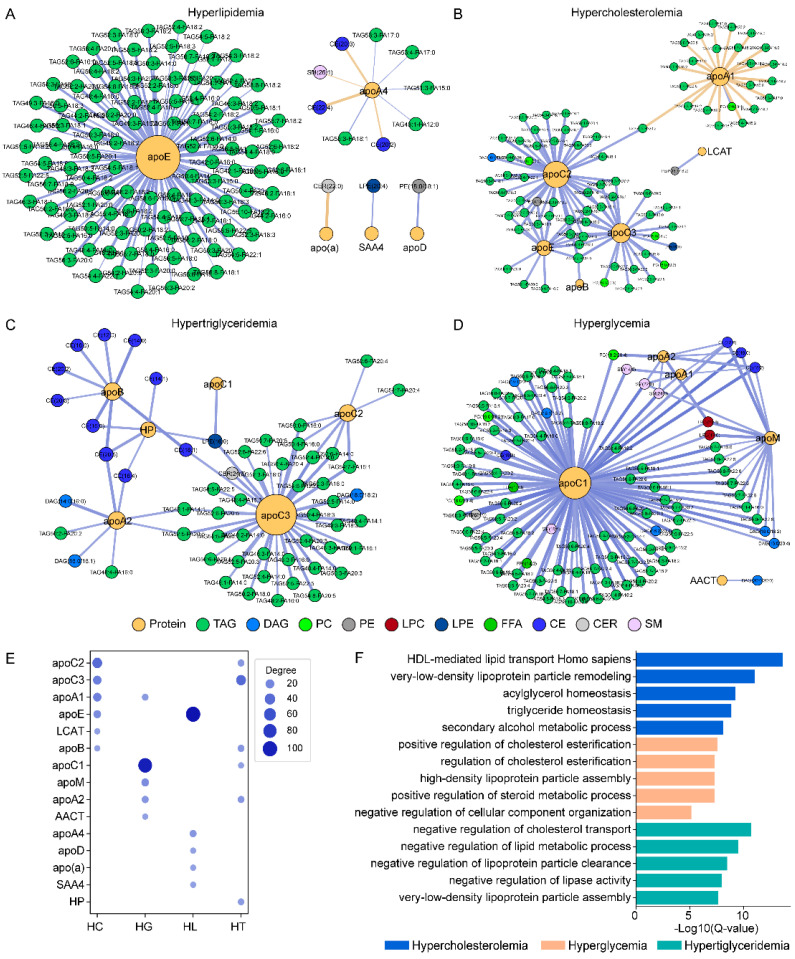
Topological networks constructed based on correlations between lipid and protein concentrations. Networks were constructed for (**A**) hyperlipidemia (**B**) hypercholesterolemia (**C**) hypertriglyceridemia and (**D**) hyperglycemia by the selection of lipid–protein pairs based on the absolute value of Pearson correlation (r > |0.5|) and the significance of the up- or down-regulation to the control samples (*p*-value < 0.05). Proteins are shown as orange circles, and the size of the circles is proportionate to the number of correlating lipid species, colored by lipid classes. (**E**) Comparison of the number of lipid species correlated with hub proteins in each group. The protein-circle sizes reflect the number of lipid species correlated with each protein. (**F**) The top-5 biological pathways and processes that were uniquely enriched based on the sets of hub proteins in the networks of hypercholesterolemia, hyperglycemia, and hypertriglyceridemia samples.

To explore the differences in the protein–lipid association among metabolic conditions, we conducted a topological analysis of protein–lipid correlation networks (Figure 4). To build the networks, we used all strongly correlating lipid–protein pairs selected in each sample group based on significant up- or down-regulation compared to the control samples (*p*-value < 0.05) and strong Pearson correlations of r > |0.5| with corresponding *p*-value < 0.002 (Supplementary Table S6).

The HL group samples gave a topological network where apoE appeared with the largest sub-network of 111 positively correlating TAG species (Figure 4A). A separate sub-network of CE, SM, and TAG species was around apoA4, including long-chain CE(20:2), CE(22:4), CE(20:3), and SM(26:1). There were also strong positive correlations between apoD and PE(18:0/18:1) and between SAA4 and LPE(10:4), as well as a negative correlation between apo(a) and CER(22:0).

The network analysis performed for the HC group revealed apoC2 as a hub surrounded by a sub-network of 37 TAGs, many of which also connected with apoC3 and apoE (Figure 4B). Interestingly, TAG(52:2-FA16:0) was connected to apos B, C2, C3, and E, while PC(16:0/18:1) to both apoA1 and apoC2. For the HT group, the protein–lipid network revealed apoC3 as the central protein associated with various TAG, DAG, LPE, CER, and CE species (Figure 4C), and some of the TAGs were shared by apoA2 or apoC3. ApoB and HP showed strong positive correlations (r = 0.50–0.65, *p*-value < 0.005) with ten CE species, and some also connected to apos A2, C3, and C1. The most unique feature of the HG group (Figure 4D) was the high number of species around apoC1 from various lipid classes including TAG, CE, CER, DAG, SM, PC, LPC, and FFA. ApoC1 also shared PC, LPC, and SM species with apoA1 and apoA2. The latter two both correlated positively with the same set of CEs and SMs. Interestingly, several DAG, CE, LPC, and SM species around apoM in the HG network were also connected to apos C1, A1, and A2.

### 3.5. Pathway Enrichment Analysis Based on Protein Data

In this study, we investigated the panel of 20 apolipoproteins and proteins involved in LDL, HDL, and VHDL remodeling. Since all 20 proteins measured in our study have defined functions in lipid metabolism, pathway enrichment analysis can uncover specific metabolic processes dysregulated in different sample categories. Furthermore, the biological functions defined for apolipoproteins allow us to link lipids to metabolic pathways through the constructed protein–lipid networks.

The enrichment analysis was performed as described in the Methods section using the sets of proteins that strongly correlated (r > |0.5|) with the highest number of lipid species in each sample group (Figure 4E,F, Supplementary Table S7). Since a different set of proteins emerged in each metabolic group, the output of the pathway enrichment analysis was expected to be different as well. For the HC group, apos A1/B/C2/C3/E and LCAT were uniquely associated with HDL-mediated lipid transport, very-low-density lipoprotein particle remodeling, acylglycerol homeostasis, and triglyceride homeostasis. For the HT group, apos A2/B/C1/C2/C3 was related to negative regulation of cholesterol transport and lipoprotein particle clearance. For the HG group, A1/A2/C1/M and AACT indicated the cholesterol esterification and HDL particle assembly. Several other pathways were common to all sample groups, including signal transduction, cholesterol homeostasis, and cholesterol transport. Nonetheless, the pathways that were unique to a metabolic condition can be indirectly linked to a set of lipids through the same set of proteins.

### 3.6. Data-Driven Parameter Screening and Artificial Neural Network Analysis

Recent studies have demonstrated the power of the deep learning approach to accurately predict dyslipidemic conditions. However, in most cases, only one condition, e.g., hyperlipidemia, or combined conditions as a single “dyslipidemia” condition, were investigated [52,53,54]. Here, we explored the feasibility to build an ANN model to differentiate all five sample categories (HC, HG, HL, HT, and NL) based on a data-driven selection of both lipid species and proteins.

**Figure 5:**
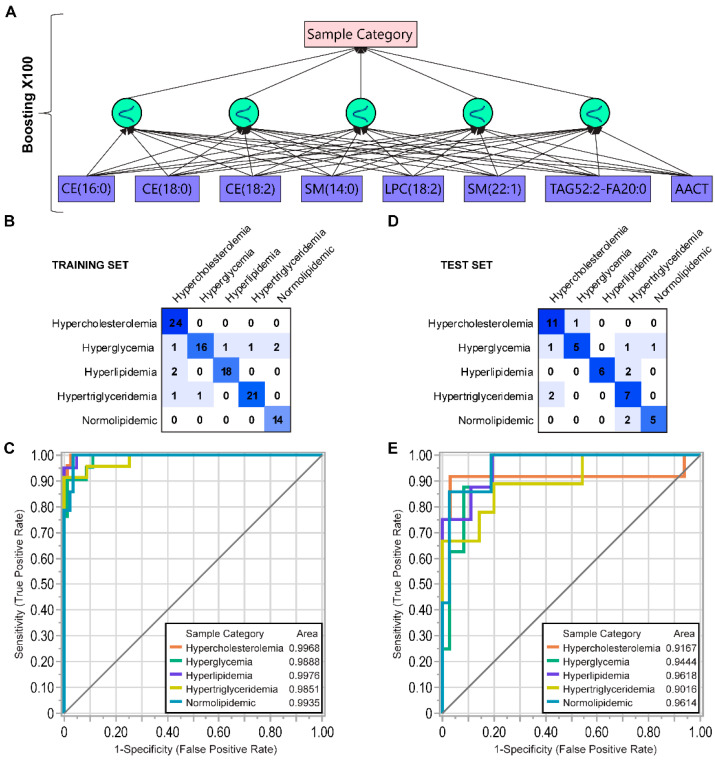
Summary of ANN machine-learning predictive modeling for metabolic conditions. (**A**) MLP neural network diagram with 8 predictors and five Gaussian activation function nodes in the hidden layer. Confusion matrix and ROC curve for the training set (**B**) and (**C**), and test set (**D**) and (**E**).

First, we randomly split the 146 samples into a training set (*n* = 102, 70%) and a test set (*n* = 44, 30%). The training set included 26 HC, 20 HG, 18 HL, 23 HT, and 15 NL samples. Then, we conducted 100 repeated cycles of the predictor selection using a bootstrap forest-based Predictor Screening algorithm implemented in JMP software. The top 20 most frequently selected variables were prioritized as the predictor pool (*p*) to build ANN models. The selected 20 predictors included 2 proteins (AACT and apoC1) and 18 lipids (Supplementary Table S8). By evaluating different types of the hidden layer and ANN architecture, we found that an optimal ANN performance can be achieved by using a fully-connected multilayer perceptron (MLP) network [55] with five Gaussian activation function nodes in the hidden layer (Figure 5A).

To determine the set of predictors that gave the highest ANN model performance, we used a step-wise systematic evaluation of all 20 predictors. The predictors P = {p_1, …, p_20} were sorted based on their relative contribution estimated by the predictor screening algorithm. Step 1, we built ANN models by using the first predictor, p_1 as “leading predictor”, and its combinations with every other predictor p_i∈P,i ∈ [2,20]. Each resulting model was evaluated in terms of the accuracy for the training and test sets (Supplementary Figure S4). The predictor combination that provided a model with the highest accuracy was kept for the next step. In step 2, a third predictor was added to the predictor set, and as in step 1, the model construction and evaluation were repeated. For the given leading predictor, this process was repeated until the addition of one more variable did not improve the model accuracy. Then, the second and each consecutive predictor (e.g., p_2, … p_20) was selected as a “leading predictor” and steps 1 and 2 were repeated. After each cycle, the models were compared based on overall model accuracy and the area under the receiver operating characteristics (ROC) curve (AUC) by each sample group. Increasing the number of predictors improved model accuracy both for the training and for the test set, but it approached a plateau at 8 predictors, in the range of 0.90–0.95 and 0.71–0.79, respectively. A total of nine models with 8 predictors were constructed. In terms of inclusion frequency, the 8-predictor combinations included 2 proteins AACT > apoC1; 4 SMs, SM(14:0) > SM(22:1) > SM(22:0) > >SM(16:0); 4 CEs, CE(18:0) > CE(18:2) > CE(16:0) > CE(20:2); 2 DAGs, DAG(18:0/18:2) > >DAG(14:0/16:0); 3 TAGs; TAG(52:2) > >TAG(51:2) > TAG(52:7); and 2 LPCs, LPC(18:2) > >LPC(18:0); HCER(24:0) and CER(24:0). The combinations of the most frequently used predictors gave an accuracy of 0.92–0.95 derived from the confusion matrix (Figure 5B) and the areas under the ROC curves (AUC) > 0.98 (Figure 5C). For the test set, the overall accuracy was 0.74–79 (Figure 5D), and the ROC AUC was > 0.90 for all sample groups (Figure 5E). The maximum accuracy was achieved for the training (0.96) and test (0.80) sets using the following 8 variables AACT, SM(14:0), SM(22:1), CE(18:0), CE(18:2) CE(16:0), LPC(18:2), and TAG(52:2).

## 4. Discussion

In the present study, we applied combined targeted lipidomics and proteomics approaches and gained comparative insights into multiple metabolic disorders. The correlation analysis of protein and lipid concentrations allowed us to create protein–lipid interaction networks that provided new insights into functional and physical associations between lipid species and apolipoproteins in different metabolic groups. For biological interpretation of our data, we rely on established theories of lipid homeostasis [56,57,58,59,60,61]. The lipid and protein compositions, as well as the relative particle numbers of HDL, LDL, and VLDL particles, are the result of their excretion rate from cells followed by extracellular remodeling. The continuous exchange of lipid species and proteins among all lipoprotein particles and their in vivo environment leads to a dynamic equilibrium concentration of individual lipid species and proteins in a fasting state. Therefore, lipid species and protein concentrations, measurable in whole plasma or serum samples, collectively characterize the lipid homeostasis of each individual person.

The HDL, LDL, and VLDL lipid species composition is the result of interconnected intracellular lipid synthesis pathways [62,63]. In our data, the interconnection of lipid pathways is evidenced by the concerted up- or down-regulation of lipid class concentrations (Figure 1A). For example, elevated TAG levels along with CERs and PEs were attributes of both HT and HL, but to a lower extent of HG or HC samples. This observation suggests a unique co-regulation of the TAG, CER, and PE de novo synthesis pathways in HT and HL patients. As another example, the upregulation of both CE and SM levels indicates an interplay between CE and SM synthesis pathways in HC and HL but not in other groups (Figure 1A). Additional evidence of interconnected lipid synthesis pathways was the corresponding abundance of DUFA and PUFA species within PC and PE classes, including plasmalogen PEs (Figure 2A,C). For instance, the overall decreased abundance of FA(18:2) across HC, HT, HL, and HG groups corresponded with an increased abundance of FA(20:4)-containing species (Figure 2B). The abundance shifts of FA(20:4)-containing species also corresponded with the increased abundance of shorter-FA-chain SM(14:0) and SM(18:0) species (Figure 2D).

The plasma lipidome also reflects the activity of intra- and extracellular lipases that hydrolyze PCs, PEs, and TAGs to LPCs, LPEs, and DAGs, respectively. The PUFA groups in PC, PE, and TAG species are frequently paired with SFA/MUFA groups on the backbone. Thus, the hydrolysis of the PUFA group produces SFA/MUFA-containing LPCs, PEs, and DAGs, and vice versa. In the non-NL groups, we observed an elevated abundance of PUFA-containing PCs, PEs, and TAGs along with the reduced abundance of PUFA-containing and increased abundance of SFA/MUFA-containing LPCs, LPEs, and DAGs (Figure 2A). Therefore, there is a higher preference for PUFA group hydrolysis from PCs, PEs, and TAGs in the non-NL groups relative to the NL group. As an example, FA(20:4)-containing species abundances are shown in Figure 2B. The reduced abundance of FA(20:4)-containing LPEs corresponded with the increased abundance of FFA(20:4) (arachidonic acid), a precursor of both pro- and anti-inflammatory eicosanoids [64].

The relative PC and LPC species composition is also affected by FC esterification, intracellularly by ACAT [65] and extracellularly by LCAT [66]; both enzymes transfer FA groups from PC to FC while producing CEs and LPCs. Increased intracellular ACAT activity was linked to a higher abundance of CE(18:1) [65], while increased extracellular LCAT activity to a higher abundance of CE(18:2) [66]. In the HT group, we found evidence for the latter, observing a decrease in the class abundance of DUFA CEs, with an increase in the class abundance of odd-chain FA and SFA/MUFA-containing CEs (Figure 2A).

The protein composition of lipoproteins fractions and sub-fractions was characterized in numerous studies [56,57,67]. On average, small HDL particles contain two apoA1 molecules while large HDL particles contain three apoA1. or 2–3 apoA1 and two apoA2 [15,31]. LDL and VLDL particles are stabilized by a single apoB molecule per particle [15]. Considering these stoichiometric and particle size/volume constraints, the combined concentration pattern of lipids, apoA1, apoA2, and apoB is expected to provide a fingerprint that corresponds with the relative particle number and size distribution of HDL, LDL, and VLDL particles.

Particle concentration and distribution by size dictate total surface area for interaction with other exchangeable apos, i.e., C1, C2, C3, D, E, and M. These proteins are in dynamic exchange among HDL, LDL, and VLDL particles. The exchange is affected by the surface affinity and penetrability by these apos, attenuated by phospholipid species composition [58,61]. Altogether, the relative concentration of exchangeable apos in plasma, along with those of apoA1, apoA2, apoB, and lipid species, contributes to a metabolic fingerprint that reflects the complexity of the within and between particle interactions.

The inverse correlation between apoA1 (or HDL particle number) and TAG concentrations in plasma is widely reported [12]. It is generally explained by the concerted actions of cholesteryl ester transferase (CETP) and lipase enzymes, resulting in the TAG transfer from VLDL to HDL particles, followed by hydrolysis of HDL TAGs, and delivery of the remaining CE content to the liver by HDL and LDL particles [8]. According to our data, this pathway is up-regulated in HC and NL groups where we observed the strongest negative correlation of apoA1 and apoA2 with TAG species, despite normal or moderately elevated TAG levels overall (Figure S3A). Interestingly, these negative correlations were significant almost exclusively with odd-chain FA, DUFA, and PUFA-containing TAGs, while correlations were few with SFA/MUFA-containing TAG species. This apparent selectivity of the TAG lowering function of HDL corroborates studies showing the TAG lowering effect of diets rich in n-3 omega fatty acids [66,68].

In theory, lipid–protein pairs are expected to correlate positively when they are simultaneously involved in the formation of a pool of particles that are similar in size. In other words, at similar core volume and surface area, both protein and lipid concentration are a function of particle number, thus the average protein and lipid concentrations show linear correlation. ApoB-containing LDL and VLDL particles collectively carry more TAG and CE molecules in plasma than apoA1-containing HDL particles. Therefore, some degree of positive correlation of TAG and CE species with apoB is expected. During extracellular remodeling, if the concentration of the lipid class and the abundance of specific species within the class change at the same time, the correlation of specific TAG or CE species with apoB may vary. The strongest apoB-TAG correlations were observed in the HC and HG group (Figure 4 and Figure S3A), in particular with higher abundant PUFA-containing TAGs (Figure 2). In the HT group, apoB also correlated strongly with CEs (Figure 4 and Figure S3A), mostly with higher abundant SFA/MUFA and DUFA-containing CEs (Figure 2).

The number of apos C1, C2, C3, and E per particle is higher on LDL/VLDL (apoB containing) than on HDL (apoA1 containing) [56,57,67]. Since LDL and VLDL particles also carry more TAGs, the increase in the LDL/VLDL particle numbers corresponds with the increases in both exchangeable apos and TAG species concentrations. In support, we found significant correlations of exchangeable apos with apoB (Figure S3B), and similarly of TAG and CE species with apoB (Figure S3A). As expected, we observed positive concentration correlations of many lipid species with exchangeable apos as well, mainly TAGs with apos C1, C2, C3, and E (Figure 4 and Figure S3A). We also found that the number and relative strength of correlations were unique to each sample group. In the HC and HT groups, apoC2 and apoC3 correlated with TAG species, but there were fewer and weaker correlations in HT (Figure 4 and Figure S3A). In the HL group, only apoE correlated strongly with TAGs. In the HG group, apoC1, C2, and apoC3 correlated strongly with nearly all monitored TAG species, however very few and weaker correlations were found between apoE and TAG species.

The abovementioned differences in protein and lipid concentrations and strengths of correlations observed for different donor groups can be used as evidence of differences in metabolic remodeling pathways. Some of these pathways can be identified through pathway enrichment analysis by using the sets of proteins whose concentration was the most significantly changed in dyslipidemic samples as compared to the NL group (Figure 4D). In HC, apos A1/B/C2/C3/E and LCAT proteins were associated with the dysregulation of HDL-mediated lipid transport, very-low-density lipoprotein particle remodeling, acylglycerol homeostasis, and triglyceride homeostasis. In HG, apos A1/A2/C1/M and AACT were linked to cholesterol esterification and HDL particle assembly. In HT, apos A2/B/C1/C2/C3 were related to negative regulation of cholesterol transport and lipoprotein particle clearance.

The concerted up- and down-shifts in lipids and protein concentrations and the relative strength of correlations are fingerprints of intertwining metabolic processes and functions for different metabolic categories. However, the construction of a predictive statistical model to evaluate the differences among complex lipidomic and proteomic fingerprints is a challenge. Traditional multivariate analysis tools, such as partial least square, principal component, and stepwise logistic regression are insufficient for the prediction of multi-level outcomes, especially in the case of a great number of intercorrelating measures [55]. To overcome these limitations, we turned to a deep learning ANN approach preceded by systematic data-driven predictor screening. The resulting ANN models with the highest accuracy contained several CEs, SMs, TAGs, and DAGs as significant classification factors. Interestingly, SM(14:0) was the strongest predictor among SMs, consistent with the ANOVA analysis, which showed its greatest increase in the HG group when other SMs were reduced. The odd-chain FA15:0-containing TAG(51:2) also emerged as a top marker, consistent with its variation in a wide range of 0.8–2.3 fold in all four non-NL groups. Similar significant markers were LPC(18:2) and LPC(18:0), probably due to their link with obesity and type 2 diabetes [69,70]. In addition, AACT emerged as the strongest protein marker. AACT is a serine protease inhibitor and inflammatory marker, also known as SerpinA3. We found that it was elevated the most in the HG group. Another protein that emerged as a putative marker was apoC1, a potent CETP inhibitor and LCAT activator, which was reduced in HG while increased in the other four groups.

## 5. Conclusions

In this study, we applied targeted lipidomics and proteomics to the same human serum samples to determine molecular characteristics of different metabolic conditions. The quantitative data-driven analysis of absolute concentration differences and concentration correlations allowed us to establish protein–lipid connectivity networks unique to each sample category and link them to defined metabolic pathways. These data also suggest the changes in the composition of HDL, LDL, and VLDL particles under different pathological conditions. The integration of larger sample sets combined with detailed follow-up experimental studies is needed to further validate and refine the condition–protein–lipid associations observed in this work. Furthermore, inclusion of phenotypic characteristics, such as gender, age, race, body mass index (BMI) and other parameters, may further inform lipid and apolipoprotein-based biomarker discovery in metabolic disorders. Nonetheless, our study demonstrates the existence of unique molecular fingerprints for each condition that can be uncovered through systematic evaluation of proteomics and lipidomics profiles. Leveraging the power of the machine-learning approach, we demonstrated the feasibility of defining a small set of molecular features for simultaneous categorization of each metabolic condition investigated in this work. Together, the application of our approach may improve molecular classification of lipid metabolism-related chronic diseases to inform new effective individualized therapeutic interventions.

## Figures and Tables

**Table 1:** Comparison of lipid class concentrations for the metabolic groups. The average lipid concentrations (min/max) were calculated by summing individual species concentrations. One-way ANOVA test was used to determine statistically significant differences between sample groups (*p*-value < 0.05 was considered significant).

Lipid Class	N	Mean Class Concentration (nmol/mL)					*p*-Value	FDR-Adjusted *p*-Value
		HL (*n* = 28)	HC (*n* = 36)	HT (*n* = 32)	HG (*n* = 29)	NL (*n* = 21)		
CE	22	6293.02 (3983.87/ 9655.85)	6582.15 (2785.74/ 11914.27)	4652.47 (2921.34/ 6470.10)	3628.28 (1493.49/ 8586.73)	3902.51 (2913.87/ 5032.15)	<0.0001	<0.0001
CER	6	11.66 (6.31/ 26.26)	11.74 (4.52/ 17.16)	14.98 (5.58/ 101.21)	9.97 (2.03/ 25.18)	7.55 (4.53/ 15.77)	<0.0001	<0.0001
DAG	17	112.60 (47.03/ 166.63)	66.47 (16.74/ 175.28)	91.61 (31.44/ 144.29)	53.65 (10.23/ 127.48)	43.65 (14.24/ 111.77)	<0.0001	<0.0001
FFA	23	1631.17 (579.04/ 3571.34)	1422.70 (477.73/ 3878.03)	1541.25 (538.41/ 2572.79)	1146.13 (435.13/ 2406.97)	775.00 (361.17/ 1506.86)	<0.0001	<0.0001
HCER	4	4.59 (2.42/ 11.78)	5.04 (1.72/ 10.58)	6.58 (2.05/ 53.38)	4.96 (1.60/ 14.58)	3.93 (2.80/ 7.66)	0.0402	0.0442
LPC	9	1155.03 (588.88/ 2091.95)	1043.70 (354.47/ 2142.20)	834.88 (44.22/ 1328.24)	611.82 (253.96/ 1220.26	637.43 (471.16/ 870.45)	<0.0001	<0.0001
LPE	4	6.01 (3.77/ 10.20)	4.84 (1.51/ 8.64)	5.65 (2.53/ 9.59)	4.74 (1.77/ 11.71)	5.67 (3.00/ 11.70)	0.0482	0.0482
PC	22	1935.99 (1411.48/ 3429.96)	1943.40 (789.95/ 2986.54)	1613.68 (107.25/ 3080.28)	1333.21 (625.37/ 2998.97)	1565.61 (1136.61/ 2293.60)	<0.0001	<0.0001
PE	20	101.81 (24.89/ 162.07)	79.43 (19.79/ 129.12)	76.98 (3.95/ 126.43)	69.64 (19.03/ 149.40)	66.83 (21.74/ 161.89)	0.0013	0.0016
SM	12	643.17 (449.83/ 961.88)	750.77 (289.47/ 1240.61)	516.03 (47.06/ 766.77)	498.27 (289.84/ 909.32)	473.00 (331.12/ 659.44)	<0.0001	<0.0001
TAG	435	3225.76 (1931.14/ 7599.63)	1651.70 (343.76/ 5488.81)	3121.76 (1308.20/ 7254.68)	1464.9 (297.58/ 4951.11)	1272.10 (415.40/ 4257.05)	<0.0001	<0.0001

**Table 2:** Comparison of protein concentrations for the metabolic groups. Average of measured protein concentrations (min/max). One-way ANOVA test was used to determine statistically significant differences between sample groups (*p*-value <0.05 was considered significant).

Protein	Mean Class Concentration (nmol/L)					*p*-Value	FDR-Adjusted *p*-Value
	HL (*n* = 28)	HC (*n* = 36)	HT (*n* = 32)	HG (*n* = 29)	NL (*n* = 21)		
apoA1	42083.17 (12188.1/ 66169.25)	56483.08 (25218.02/ 95088.75)	44540.11 (32114.84/ 70397.61)	37341.74 (21008.5/ 51743.20)	43776.14 (27504.08/ 75445.27)	<0.0001	<0.0001
AACT	31373.50 (7716.27/ 113819.02)	34964.14 (5340.99/ 72572.32)	32233.06 (18656.83/ 54612.17)	46986.16 (9633.38/ 101862.96)	27568.85 (21209.76/ 40478.80)	0.0022	0.0039
apoA2	37812.64 (5204.16/ 68486.21)	41216.74 (5033.16/ 58668.71)	36878.18 (22066.13/ 73083.55)	27421.17 (7478.85/ 50263.66)	39002.86 (28271.1/ 51640.55)	<0.0001	<0.0001
apoA4	1975.83 (174.47/ 5096.85)	2061.60 (177.50/ 5947.96)	2212.38 (653.78/ 4666.87)	2146.20 (1095.56/ 4497.41)	1425.91 (855.8/ 2412.68)	0.0066	0.0110
apoB	2135.44 (438.21/ 4035.01)	2207.65 (741.96/ 3748.06)	1760.61 (894.51/ 3366.66)	1360.11 (682.29/ 3038.84)	1220.63 (801.20/ 2503.91)	<0.0001	<0.0001
apoC1	11107.72 (2495.45/ 17306.72)	13685.27 (5109.03/ 43096.16)	10828.05 (5094.76/ 16227.18)	7486.51 (2593.07/ 17171.94)	8227.14 (5379.98/ 13462.97)	<0.0001	<0.0001
apoC2	6016.09 (1563.75/ 11102.30)	5443.62 (1401.34/ 11685.60)	5535.84 (1740.988/ 9993.20)	3590.02 (369.23/ 11300.71)	2998.99 (1162.95/ 7131.60)	<0.0001	<0.0001
apoC3	15971.44 (1169.71/ 27413.37)	14438.28 (1479.65/ 27848.45)	15485.42 (6080.41/ 30489.17)	9869.37 (1282.52/ 23024.64)	8056.58 (4673.48/ 18165.67)	<0.0001	<0.0001
apoD	2159.77 (314.833/ 11649.68)	2370.71 (908.66/ 8286.79)	1995.23 (1164.22/ 3638.30)	2121.42 (1256.00/ 3785.67)	2322.35 (1405.64/ 3247.54)	0.2224	0.2694
apoE	1849.06 (428.23/ 4051.86)	1912.59 (56.99/ 4304.84)	1784.22 (1034.24/ 3063.06)	1512. 58 (703.88/ 3917.80)	1149.86 (658.55/ 2423.09)	0.0014	0.0028
apoM	910.14 (249.59/ 2211.54)	986.90 (240.97/ 1623.97)	819.74 (449.36/ 1377.66)	684.93 (340.00/ 1295.73)	910.80 (534.75/ 1446.49)	0.0007	0.0016
CETP	32.15 (3.86/ 114.79)	46.06 (10.53/ 202.26)	43.04 (9.40/ 216.23)	24.63 (4.68/ 73.84)	33.36 (14.47/ 74.90)	0.2490	0.2767
HP	22627.85 (2830.18/ 49552.20)	26019.40 (2791.24/ 73051.78)	30434.21 (5063.66/ 65007.75)	37418.85 (5029.59/ 96991.16)	17136.29 (2724.09/ 39682.59)	0.0011	0.0025
LCAT	104.62 (38.38/ 169.55)	111.42 (35.77/ 216.33)	106.36 (25.29/ 176.85)	81.85 (25.05/ 154.67)	76.81 (57.80/ 123.69)	<0.0001	0.0003
apo(a)	66.42 (13.84/ 468.77)	100.22 (13.67/ 380.86)	54.40 (9.95/ 230.45)	110.40 (22.10/ 505.71)	82.53 (23.09/ 205.41)	0.0610	0.0813
PLTP	74.76 (38.26/ 134.08)	88.63 (19.33/ 150.89)	76.80 (27.86/ 126.74)	98.94 (61.72/ 195.06)	76.33 (57.17/ 113.82)	0.0226	0.0347
PON1	1462.74 (255.70/ 2981.05)	1774.69 (268.72/ 3472.60	1715.17 (579.82/ 3216.10)	1442.07 (329.14/ 3697.67)	1644.84 (747.88/ 3996.51)	0.2681	0.2823
SAA1	1032.14 (142.51/ 12268.88)	1190.87 (203.90/ 9550.01)	866.73 (115.45/ 4341.76)	1520.89 (62.15/ 14491.09)	472.2 8(82.01/ 2027.73)	0.2290	0.2694
SAA4	2347.91 (351.09/ 4767.17)	2320.39 (765.17/ 7827.47)	2015.95 (980.35/ 4298.63)	2452.47 (561.43/ 5812.54)	1713.41 (1124.85/ 3026.67)	0.0583	0.0813
TF	12208.70 (3204.43/ 75876.58)	10557.5 8(3015.28/ 53645.74)	11204.71 (3628.40/ 64739.72)	8036.81 (2535.74/ 14123.04)	1230.71 (3922.83/ 105927.36)	0.6225	0.6225

## Data Availability

The datasets used and/or analyzed during the current study are contained within the manuscript and available from the corresponding author on reasonable request.

## Supplementary Materials

The following supporting information can be downloaded at: https://www.mdpi.com/article/10.3390/biom12101439/s1, Figure S1: Pearson correlation between apolipoproteins and lipid species did not follow the trend in significant differentiation from the NL group; Figure S2: Pearson correlation between apolipoproteins and lipid species did not follow the order of %Abundance of species in lipid classes; Figure S3: Percent proportion of correlating lipid species relative to the total number of monitored species, and correlation of apos A1 and B with other apos. (a) Percentage of the number of lipid species that significantly correlated with apolipoproteins in CE, PC, and TAG sub-classes (SFA/MUFA, PUFA, DUFA, or odd-chain FA) for HC, HT, HL, and HG. Stacked bar graphs indicate Pearson correlations r > 0.3 (blue), r > 0.5 (red), r < −0.3 (green), and r < −0.5 (brown) (regardless of up-or down-regulation relative to NL, as in Figure 4). (b) Pearson correction coefficient of apoA1 and apoB with exchangeable apos; Figure S4: Optimization of predictors combinations using 3 to 14 variables from the list of top 20 predictors for the final ANN model. Each ANN model was evaluated based on accuracy parameters for the training and test set; Figure S5: Lack of agreement of Pearson correlations between proteins and lipid species (*y*-axis) versus between proteins and lipid classes (*x*-axis), indicating unique information provided by correlation with lipid species; Figure S6: Various degrees of up- and down-regulation of top 20 variables in metabolic groups relative to the normolipidemic control group; Figure S7: Representative chromatograms for targeted proteomics; Table S1: List of deuterium-labeled internal standards for Lipidyzer platform; Table S2: The coefficient of variation (CV) for lipid class and proteins calculated for the quality control (QC) samples; Table S3: Means comparison analysis for lipid species and proteins; Table S4: Summary table of lipids (nmol/mL) and proteins (nmol/L) concentrations; Table S5: Lipid species and proteins significantly upregulated in different metabolic groups; Table S6: Lipid–protein correlation analysis; Table S7: Pathway enrichment analysis; Table S8: The top-20 predicted selected for the ANN modeling.
