# Supplementary material for: Integrated Quantitative Targeted Lipidomics and Proteomics Reveal Unique Fingerprints of Multiple Metabolic Conditions
Source: Biomolecules. 2022 Oct 8;12(10):1439. doi: 10.3390/biom12101439 (PMC9599481; doi:10.3390/biom12101439)
Supplement: Supplementary file 1 [file biomolecules-12-01439-s001.zip › Supplemental_Figures.pdf]

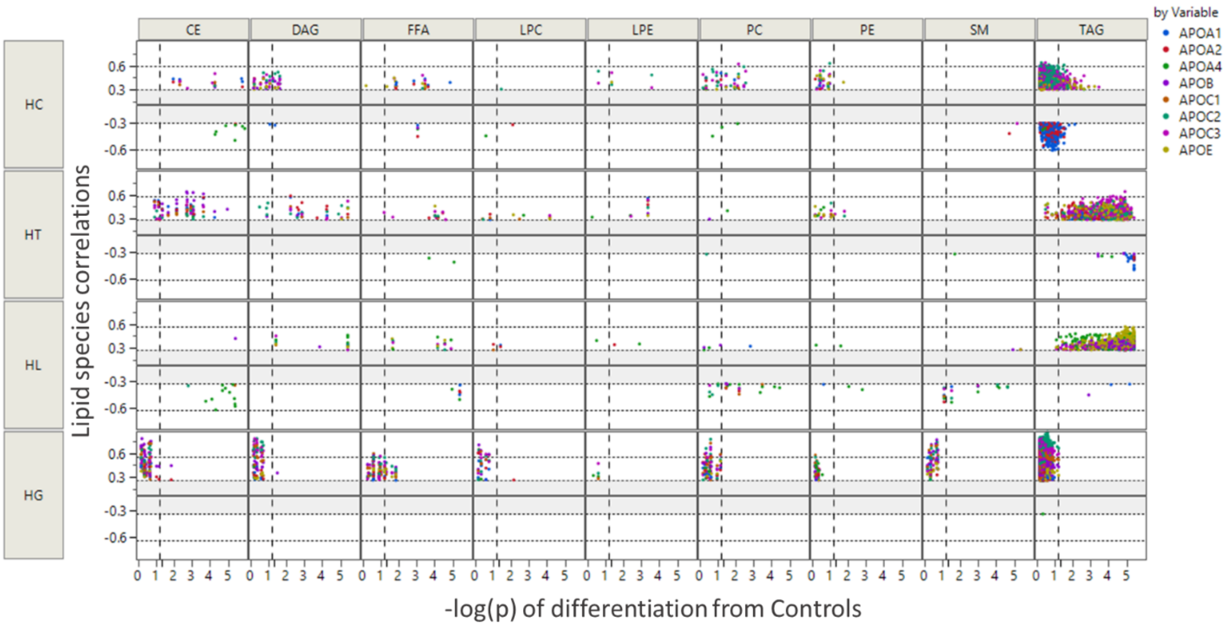

Figure S1. Pearson correlation between apolipoproteins and lipid species did not follow the trend in significant differentiation from the NL group.

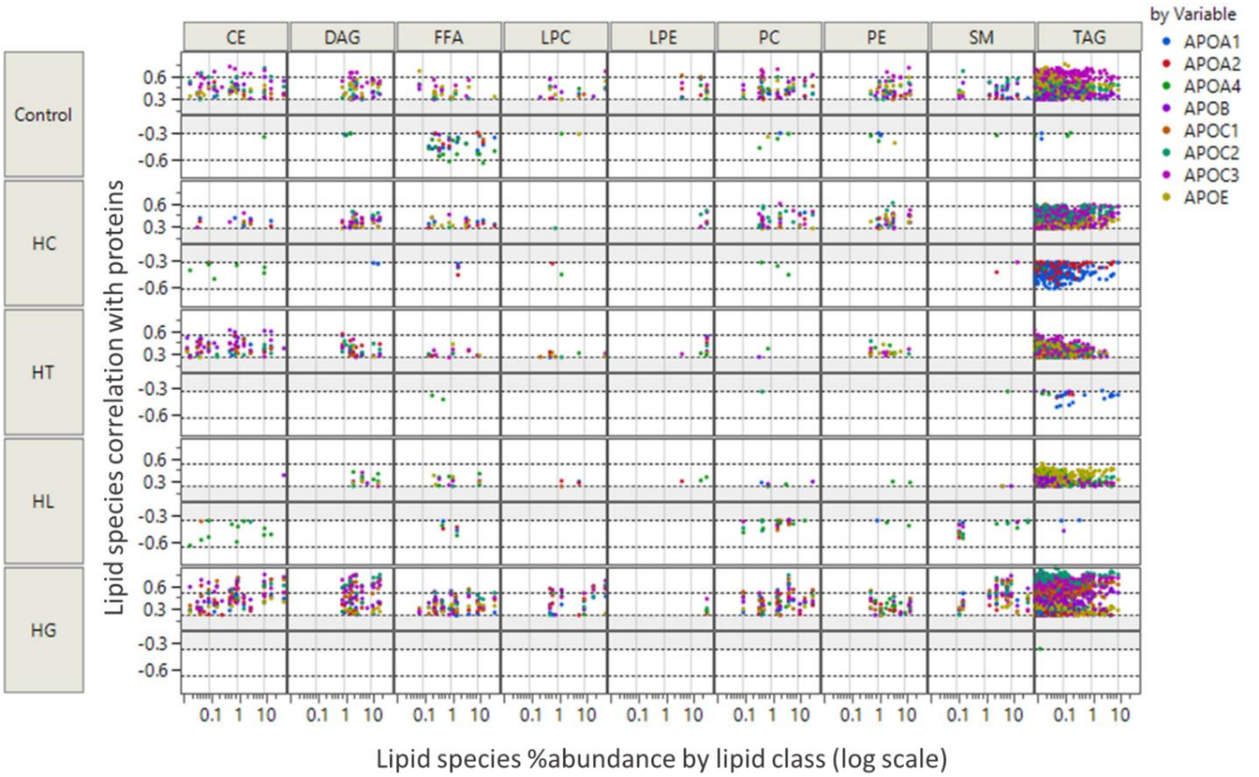

Figure S2. Pearson correlation between apolipoproteins and lipid species did not follow the order of %Abundance of species in lipid classes.

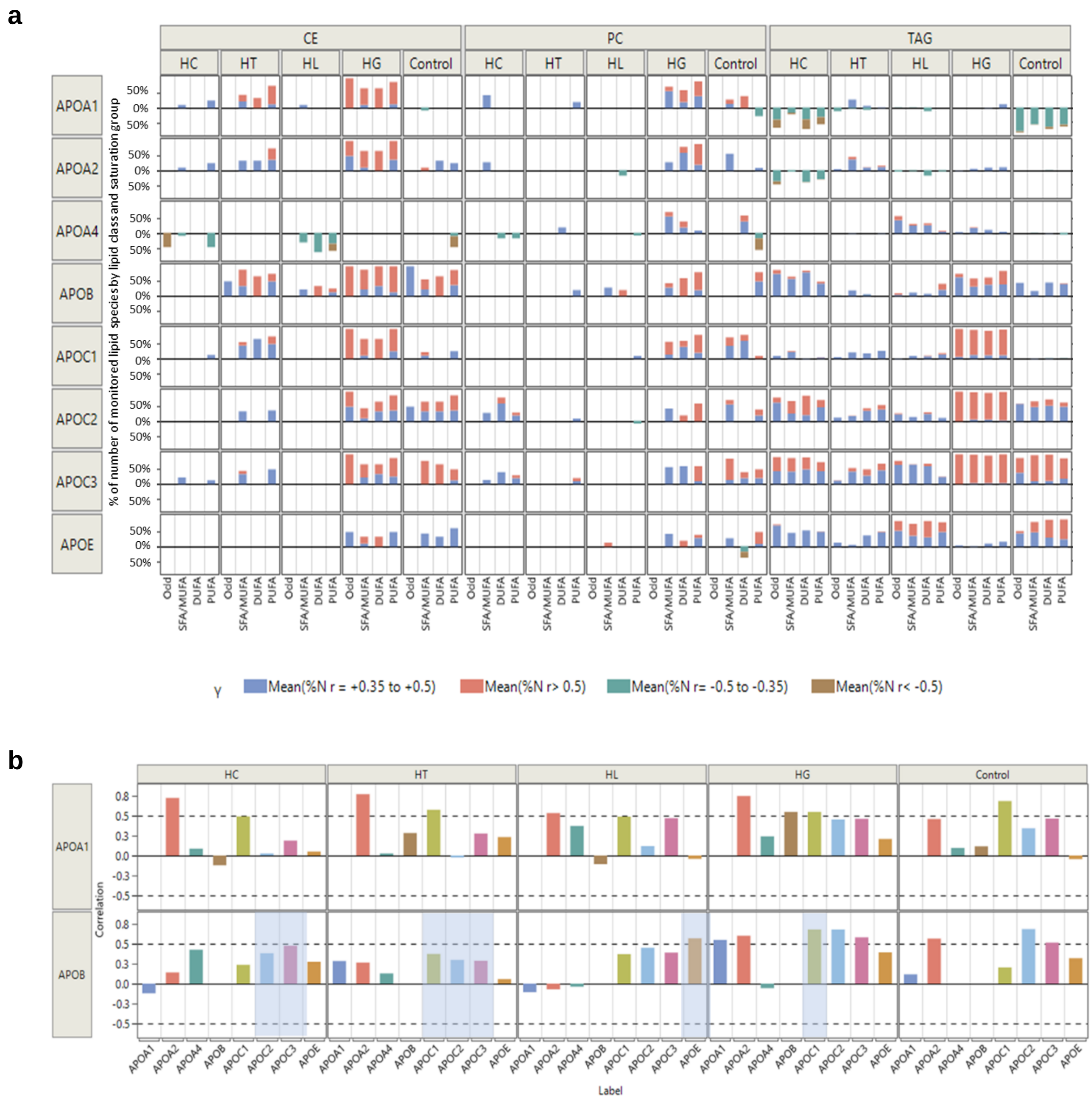

Figure S3. Percent proportion of correlating lipid species relative to the total number of monitored species, and correlation of apos A1 and B with other apos. (a) Percentage of the number of lipid species that significantly correlated with apolipoproteins in CE, PC, and TAG sub-classes (SFA/MUFA, PUFA, DUFA, or odd-chain FA) for HC, HT, HL, and HG. Stacked bar graphs indicate Pearson correlations r > 0.3 (blue), r > 0.5 (red), r < -0.3 (green) and r < -0.5 (brown) (regardless of up-or down-regulation relative to NL, as in Fig. 4). (b) Pierson correction coefficient of apoA1 and apoB with exchangeable apos.

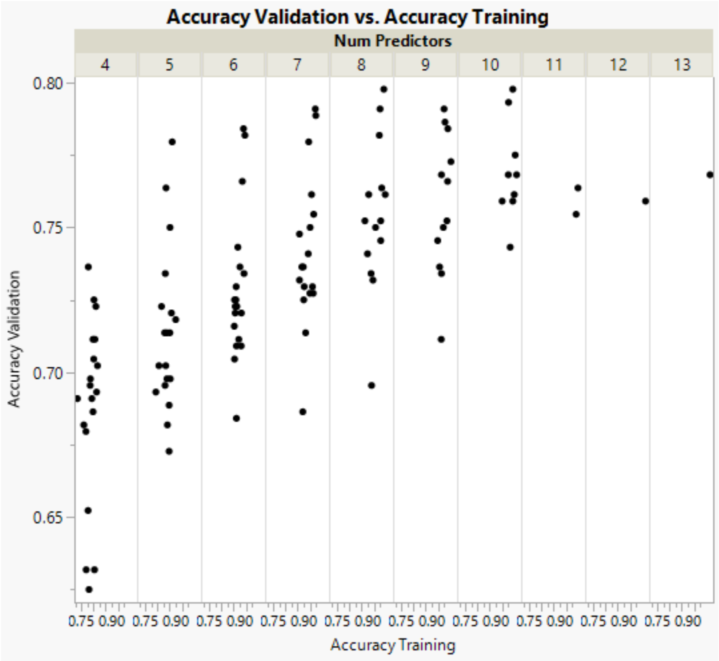

Figure S4. Optimization of predictors combinations using 3 to 14 variables from the list of top 20 predictors for the final ANN model. Each ANN model was evaluated based on accuracy parameters for the training and test set.

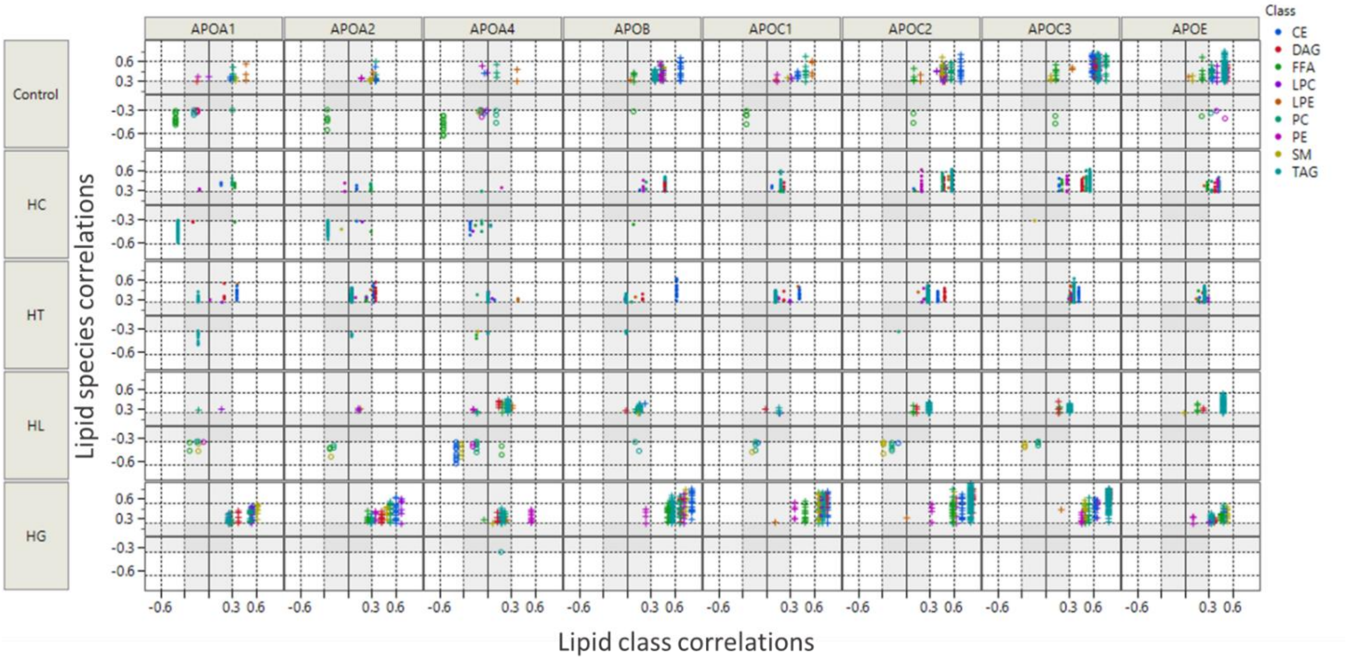

Figure S5. Lack of agreement of Pearson correlations between proteins and lipid species (y-axis) versus between proteins and lipid classes (x-axis), indicating unique information provided by correlation with lipid species.

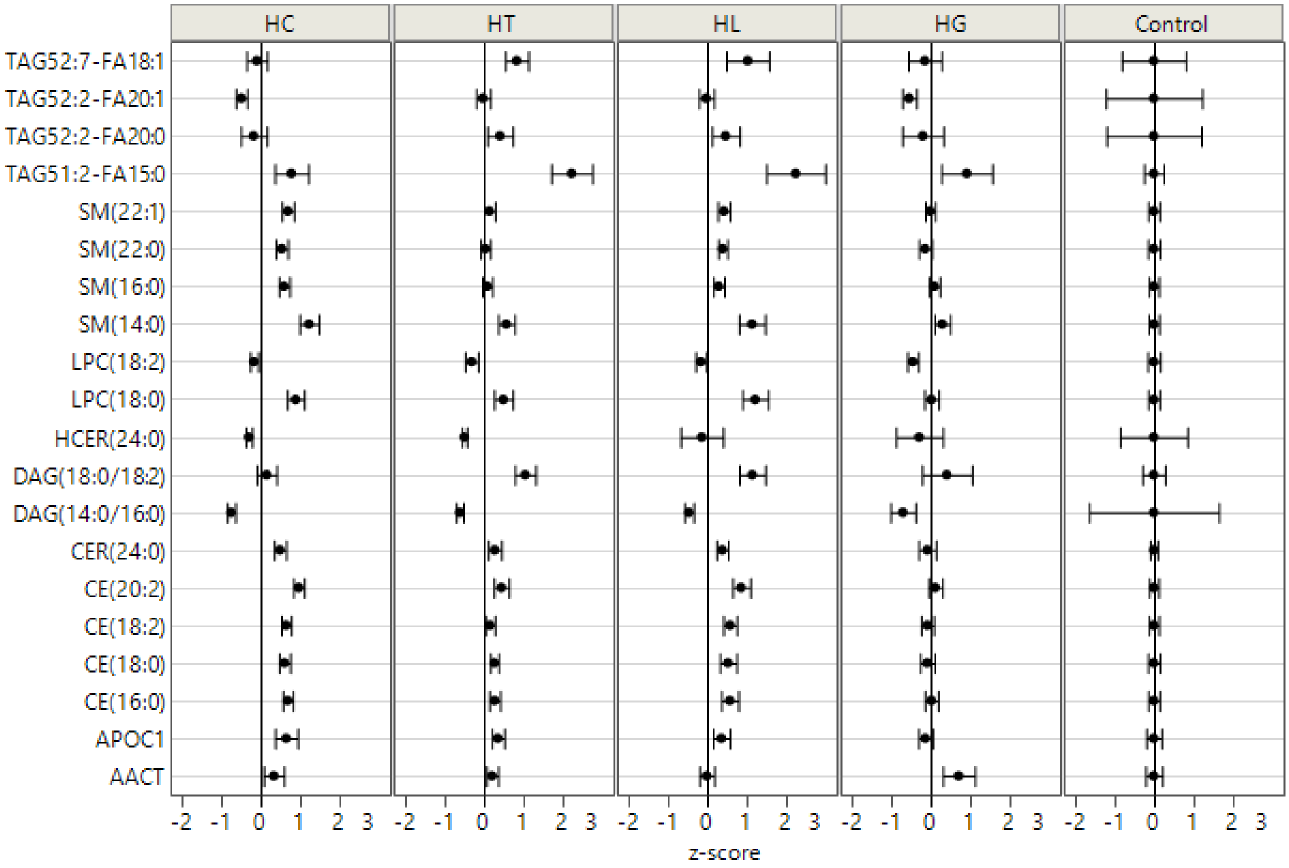

Figure S6. Various degrees of up and downregulation of top 20 variables in metabolic groups relative to the normolipidemic control group.

a

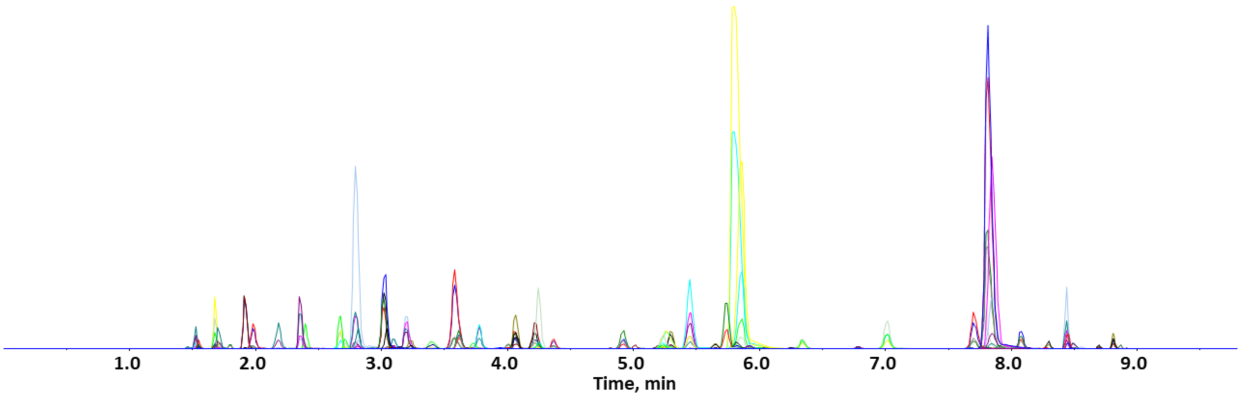

b

APOA1

Protein	Q1 Mass	Q3 Mass	Transition Name
APOA1	608.31	777.44	APOA1.ATEHLSTLSEK.+2y7.light
APOA1	608.31	664.35	APOA1.ATEHLSTLSEK.+2y6.light
APOA1	611.82	784.45	APOA1.ATEHLSTLSEK.+2y7.heavy
APOA1	611.82	671.37	APOA1.ATEHLSTLSEK.+2y6.heavy
APOA1	651.33	1063.54	APOA1.THLAPYSDELR.+2y9.light
APOA1	651.33	950.46	APOA1.THLAPYSDELR.+2y8.light
APOA1	654.84	1070.56	APOA1.THLAPYSDELR.+2y9.heavy
APOA1	654.84	957.47	APOA1.THLAPYSDELR.+2y8.heavy
APOA1	506.79	813.45	APOA1.AKPALEDLR.+2y7.light
APOA1	506.79	716.39	APOA1.AKPALEDLR.+2y6.light
APOA1	510.30	820.46	APOA1.AKPALEDLR.+2y7.heavy
APOA1	510.30	723.41	APOA1.AKPALEDLR.+2y6.heavy

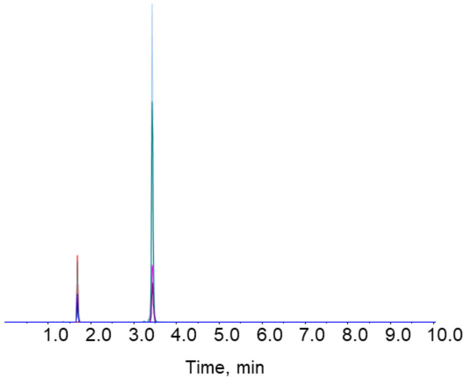

AACT

Protein	Q1 Mass	Q3 Mass	Transition Name
AACT	531.30	819.46	AACT.EIGELYLPK.+2y7.light
AACT	531.30	762.44	AACT.EIGELYLPK.+2y6.light
AACT	535.30	827.48	AACT.EIGELYLPK.+2y7.heavy
AACT	535.30	770.45	AACT.EIGELYLPK.+2y6.heavy

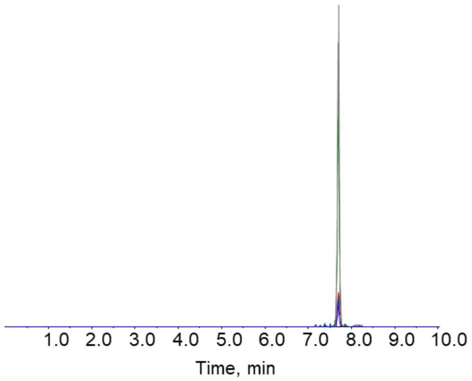

APOA2

Protein	Q1 Mass	Q3 Mass	Transition Name
APOA2	471.29	571.38	APOA2.EQLTPLIK.+2y5.light
APOA2	471.29	470.33	APOA2.EQLTPLIK.+2y4.light
APOA2	474.80	578.40	APOA2.EQLTPLIK.+2y5.heavy
APOA2	474.80	477.35	APOA2.EQLTPLIK.+2y4.heavy

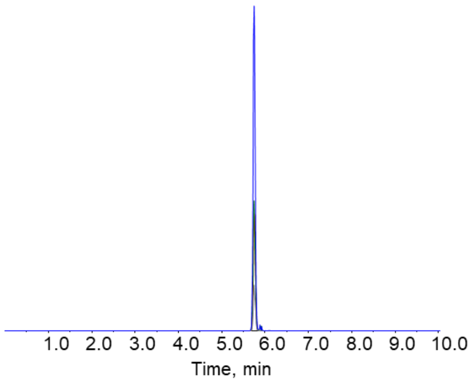

APOA4

Protein	Q1 Mass	Q3 Mass	Transition Name
APOA4	552.79	862.44	APOA4.LEPYADQLR.+2y7.light
APOA4	552.79	765.39	APOA4.LEPYADQLR.+2y6.light
APOA4	556.30	869.46	APOA4.LEPYADQLR.+2y7.heavy
APOA4	556.30	772.41	APOA4.LEPYADQLR.+2y6.heavy
APOA4	542.27	869.40	APOA4.LTPYADEFK.+2y7.light
APOA4	542.27	772.35	APOA4.LTPYADEFK.+2y6.light
APOA4	546.28	877.42	APOA4.LTPYADEFK.+2y7.heavy
APOA4	546.28	780.37	APOA4.LTPYADEFK.+2y6.heavy

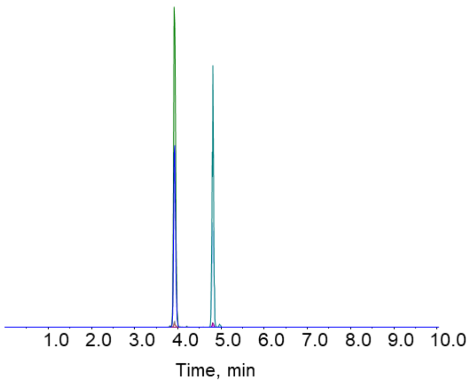

APOB

Protein	Q1 Mass	Q3 Mass	Transition Name
APOB	621.82	914.46	APOB.ATGVLYDYVNK.+2y7.light
APOB	621.82	801.38	APOB.ATGVLYDYVNK.+2y6.light
APOB	624.83	920.48	APOB.ATGVLYDYVNK.+2y7.heavy
APOB	624.83	807.39	APOB.ATGVLYDYVNK.+2y6.heavy
APOB	371.73	600.38	APOB.AAIQALR.+2y5.light
APOB	371.73	487.30	APOB.AAIQALR.+2y4.light
APOB	375.24	607.40	APOB.AAIQALR.+2y5.heavy
APOB	375.24	494.32	APOB.AAIQALR.+2y4.heavy
APOB	506.82	741.49	APOB.TGISPLALIK.+2y7.light
APOB	506.82	654.45	APOB.TGISPLALIK.+2y6.light
APOB	510.33	748.50	APOB.TGISPLALIK.+2y7.heavy

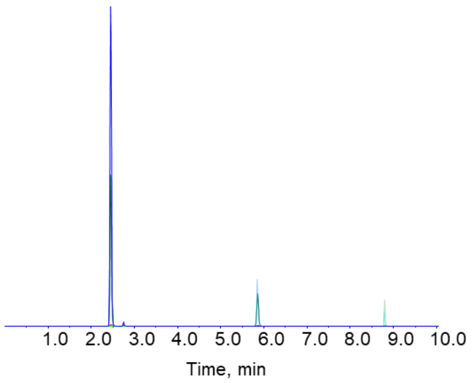

APOC1

Protein	Q1 Mass	Q3 Mass	Transition Name
APOC1	601.28	886.43	APOC1.EWFSETFQK.+2y7.light
APOC1	601.28	739.36	APOC1.EWFSETFQK.+2y6.light
APOC1	605.29	894.44	APOC1.EWFSETFQK.+2y7.heavy
APOC1	605.29	747.38	APOC1.EWFSETFQK.+2y6.heavy
APOC1	516.76	834.42	APOC1.TPDVSSALDK.+2y8.light
APOC1	516.76	620.32	APOC1.TPDVSSALDK.+2y6.light
APOC1	520.27	841.44	APOC1.TPDVSSALDK.+2y8.heavy
APOC1	520.27	627.34	APOC1.TPDVSSALDK.+2y6.heavy

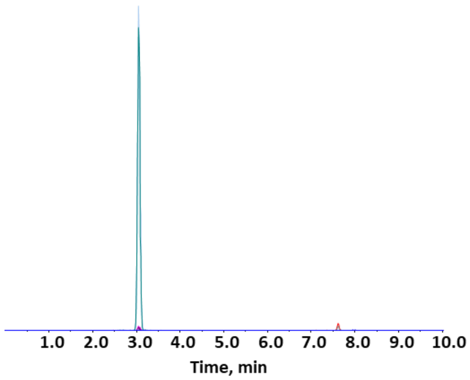

APOC2

Protein	Q1 Mass	Q3 Mass	Transition Name
APOC2	643.80	957.43	APOC2.ESLSSYWESAK.+2y8.light
APOC2	643.80	870.40	APOC2.ESLSSYWESAK.+2y7.light
APOC2	647.81	965.45	APOC2.ESLSSYWESAK.+2y8.heavy
APOC2	647.81	878.41	APOC2.ESLSSYWESAK.+2y7.heavy
APOC2	518.27	771.42	APOC2.TYLPAVDEK.+2y7.light
APOC2	518.27	658.34	APOC2.TYLPAVDEK.+2y6.light
APOC2	521.28	777.44	APOC2.TYLPAVDEK.+2y7.heavy
APOC2	521.28	664.35	APOC2.TYLPAVDEK.+2y6.heavy

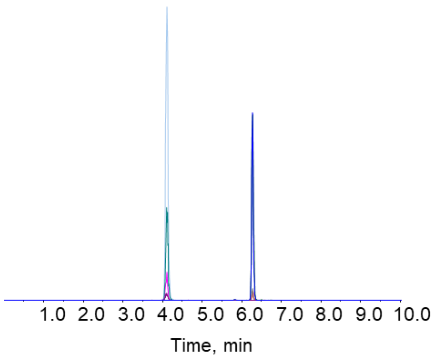

APOC3

Protein	Q1 Mass	Q3 Mass	Transition Name
APOC3	858.93	1016.51	APOC3.DALSSVQESQVAQQAR.+2y9.light
APOC3	858.93	887.47	APOC3.DALSSVQESQVAQQAR.+2y8.light
APOC3	862.43	1023.52	APOC3.DALSSVQESQVAQQAR.+2y9.heavy
APOC3	862.43	894.48	APOC3.DALSSVQESQVAQQAR.+2y8.heavy
APOC3	598.80	854.43	APOC3.GWVTDGFSSLK.+2y8.light
APOC3	598.80	753.38	APOC3.GWVTDGFSSLK.+2y7.light
APOC3	602.31	861.44	APOC3.GWVTDGFSSLK.+2y8.heavy
APOC3	602.31	760.39	APOC3.GWVTDGFSSLK.+2y7.heavy

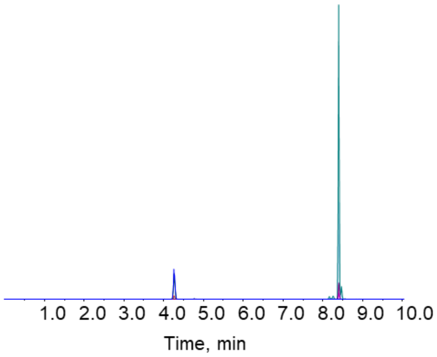

APOD

Protein	Q1 Mass	Q3 Mass	Transition Name
APOD	436.25	772.43	APOD.VLNQELR.+2y6.light
APOD	436.25	659.35	APOD.VLNQELR.+2y5.light
APOD	439.76	779.45	APOD.VLNQELR.+2y6.heavy
APOD	439.76	666.36	APOD.VLNQELR.+2y5.heavy

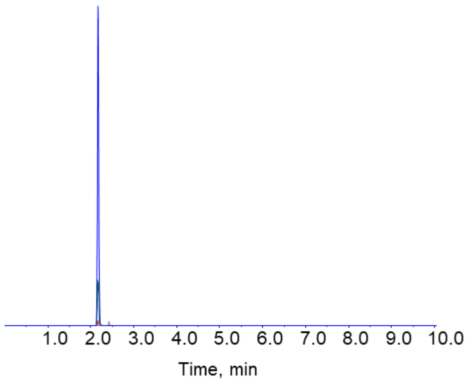

APOE

Protein	Q1 Mass	Q3 Mass	Transition Name
APOE	749.40	898.47	APOE.AATVGSLAGQPLQER.+2y8.light
APOE	749.40	827.44	APOE.AATVGSLAGQPLQER.+2y7.light
APOE	752.91	905.48	APOE.AATVGSLAGQPLQER.+2y8.heavy
APOE	752.91	834.45	APOE.AATVGSLAGQPLQER.+2y7.heavy
APOE	484.78	588.31	APOE.LGPLVEQGR.+2y5.light
APOE	484.78	489.24	APOE.LGPLVEQGR.+2y4.light
APOE	488.29	595.32	APOE.LGPLVEQGR.+2y5.heavy
APOE	488.29	496.25	APOE.LGPLVEQGR.+2y4.heavy
APOE	517.27	792.40	APOE.LQAEAFQAR.+2y7.light
APOE	517.27	721.36	APOE.LQAEAFQAR.+2y6.light
APOE	520.78	799.41	APOE.LQAEAFQAR.+2y7.heavy
APOE	520.78	728.37	APOE.LQAEAFQAR.+2y6.heavy

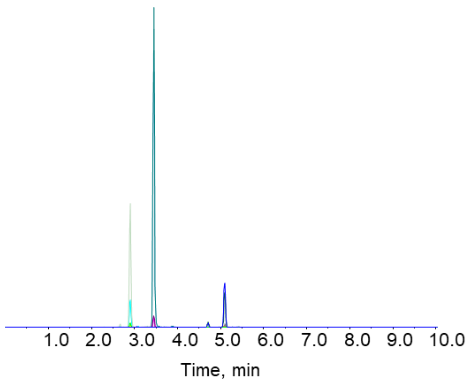

APOM

Protein	Q1 Mass	Q3 Mass	Transition Name
APOM	413.23	565.31	APOM.FLLYNR.+2y4.light
APOM	413.23	452.23	APOM.FLLYNR.+2y3.light
APOM	416.74	572.33	APOM.FLLYNR.+2y4.heavy
APOM	416.74	452.23	APOM.FLLYNR.+2y3.heavy

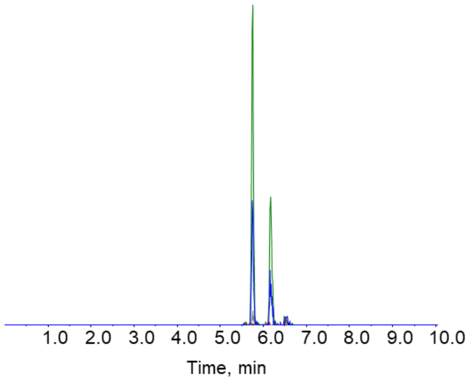

CETP

Protein	Q1 Mass	Q3 Mass	Transition Name
CETP	495.777373	788.469872	CETP.AMMLLGQVK.+2y7.light
CETP	495.777373	657.429387	CETP.AMMLLGQVK.+2y6.light
CETP	498.784278	794.483681	CETP.AMMLLGQVK.+2y7.heavy
CETP	498.784278	663.443196	CETP.AMMLLGQVK.+2y6.heavy
CETP	481.774534	863.473377	CETP.VIQTAFQR.+2y7.light
CETP	481.774534	750.389313	CETP.VIQTAFQR.+2y6.light
CETP	481.774534	622.330736	CETP.VIQTAFQR.+2y5.light
CETP	481.774534	521.283057	CETP.VIQTAFQR.+2y4.light
CETP	486.778668	873.481646	CETP.VIQTAFQR.+2y7.heavy
CETP	486.778668	760.397582	CETP.VIQTAFQR.+2y6.heavy
CETP	486.778668	632.339005	CETP.VIQTAFQR.+2y5.heavy
CETP	486.778668	531.291326	CETP.VIQTAFQR.+2y4.heavy

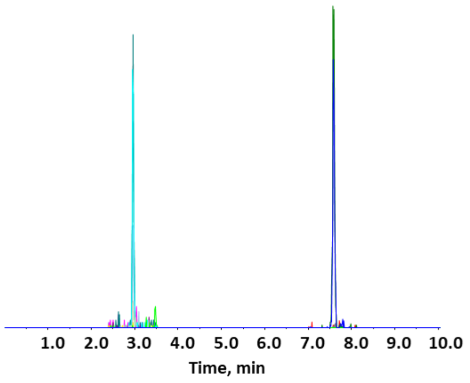

HP

Protein	Q1 Mass	Q3 Mass	Transition Name
HP	352.190302	604.304915	HP.VSVNER.+2y5.light
HP	352.190302	517.272886	HP.VSVNER.+2y4.light
HP	352.190302	418.204472	HP.VSVNER.+2y3.light
HP	357.194437	614.313184	HP.VSVNER.+2y5.heavy
HP	357.194437	527.281155	HP.VSVNER.+2y4.heavy
HP	357.194437	428.212741	HP.VSVNER.+2y3.heavy
HP	380.703046	613.330401	HP.FTDHLK.+2y5.light
HP	380.703046	512.282723	HP.FTDHLK.+2y4.light
HP	380.703046	397.25578	HP.FTDHLK.+2y3.light
HP	384.710145	621.3446	HP.FTDHLK.+2y5.heavy
HP	384.710145	520.296922	HP.FTDHLK.+2y4.heavy
HP	384.710145	405.269979	HP.FTDHLK.+2y3.heavy

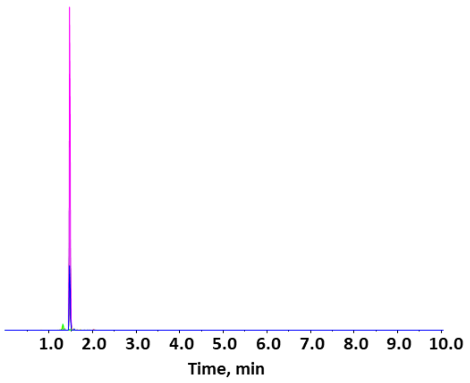

LCAT

Protein	Q1 Mass	Q3 Mass	Transition Name
LCAT	692.88079	941.516305	LCAT.SSGLVSNAPGVQIR.+2y9.light
LCAT	692.88079	669.404235	LCAT.SSGLVSNAPGVQIR.+2y6.light
LCAT	696.389372	948.533469	LCAT.SSGLVSNAPGVQIR.+2y9.heavy
LCAT	696.389372	676.421399	LCAT.SSGLVSNAPGVQIR.+2y6.heavy

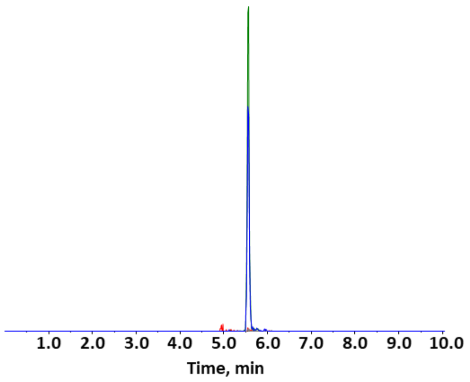

APO(a)

Protein	Q1 Mass	Q3 Mass	Transition Name
APO(a)	521.76182	721.383893	LPA.GTYSTTVTGR.+2y7.light
APO(a)	521.76182	634.351865	LPA.GTYSTTVTGR.+2y6.light
APO(a)	524.768725	727.397702	LPA.GTYSTTVTGR.+2y7.heavy
APO(a)	524.768725	640.365674	LPA.GTYSTTVTGR.+2y6.heavy
APO(a)	686.330598	877.452642	LPA.TTEYYPNGGLTR.+2y8.light
APO(a)	686.330598	714.389313	LPA.TTEYYPNGGLTR.+2y7.light
APO(a)	689.83918	884.469806	LPA.TTEYYPNGGLTR.+2y8.heavy
APO(a)	689.83918	721.406477	LPA.TTEYYPNGGLTR.+2y7.heavy

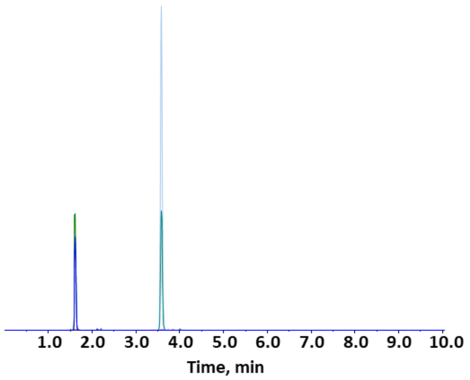

PLTP

Protein	Q1 Mass	Q3 Mass	Transition Name
PLTP	599.363713	885.540394	PLTP.AGALQLLLVGDK.+2y8.light
PLTP	599.363713	757.481817	PLTP.AGALQLLLVGDK.+2y7.light
PLTP	602.370617	891.554203	PLTP.AGALQLLLVGDK.+2y8.heavy
PLTP	602.370617	763.495626	PLTP.AGALQLLLVGDK.+2y7.heavy
PLTP	326.710674	440.261593	PLTP.VLAPPR.+2y4.light
PLTP	326.710674	369.22448	PLTP.VLAPPR.+2y3.light
PLTP	329.717578	446.275402	PLTP.VLAPPR.+2y4.heavy
PLTP	329.717578	375.238288	PLTP.VLAPPR.+2y3.heavy

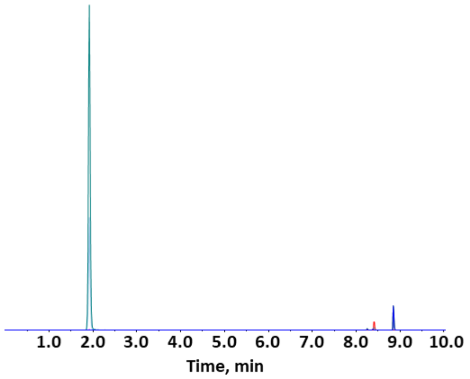

PON1

Protein	Q1 Mass	Q3 Mass	Transition Name
PON1	592.829703	943.509488	PON1.IQNILTEEPK.+2y8.light
PON1	592.829703	603.298432	PON1.IQNILTEEPK.+2y5.light
PON1	596.836802	951.523687	PON1.IQNILTEEPK.+2y8.heavy
PON1	596.836802	611.312631	PON1.IQNILTEEPK.+2y5.heavy
PON1	660.371538	1057.604057	PON1.YVYIAELLAHK.+2y9.light
PON1	660.371538	781.456664	PON1.YVYIAELLAHK.+2y7.light
PON1	663.88012	1064.621221	PON1.YVYIAELLAHK.+2y9.heavy
PON1	663.88012	788.473828	PON1.YVYIAELLAHK.+2y7.heavy

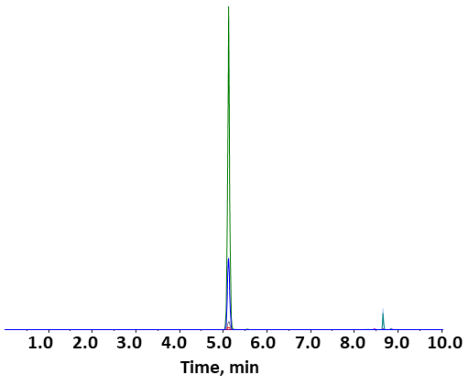

SAA1

Protein	Q1 Mass	Q3 Mass	Transition Name
SAA1	728.862597	903.453036	SAA1.GPGGVWAAEAISDAR.+2y9.light
SAA1	728.862597	761.378808	SAA1.GPGGVWAAEAISDAR.+2y7.light
SAA1	733.866731	913.461305	SAA1.GPGGVWAAEAISDAR.+2y9.heavy
SAA1	733.866731	771.387077	SAA1.GPGGVWAAEAISDAR.+2y7.heavy

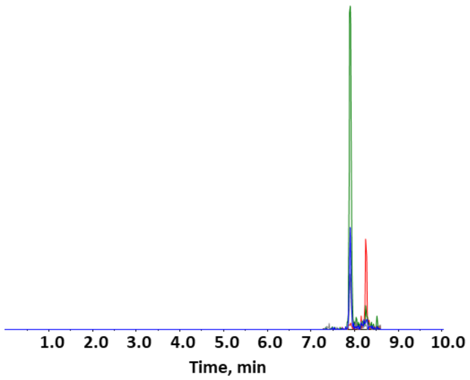

SSA4

Protein	Q1 Mass	Q3 Mass	Transition Name
SAA4	421.729595	688.377686	SAA4.GPGGVWAAK.+2y7.light
SAA4	421.729595	631.356222	SAA4.GPGGVWAAK.+2y6.light
SAA4	425.736694	696.391885	SAA4.GPGGVWAAK.+2y7.heavy
SAA4	425.736694	639.370421	SAA4.GPGGVWAAK.+2y6.heavy

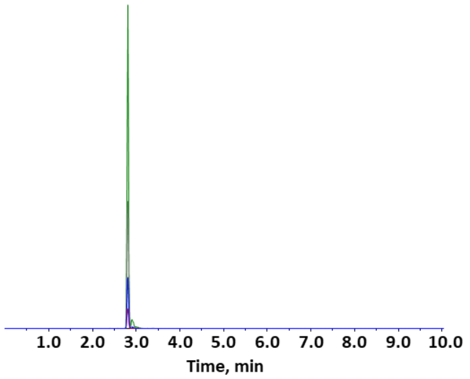

TF

Protein	Q1 Mass	Q3 Mass	Transition Name
TF	500.752932	724.351196	TF.YLGEEYVK.+2y6.light
TF	500.752932	538.287139	TF.YLGEEYVK.+2y4.light
TF	504.760032	732.365395	TF.YLGEEYVK.+2y6.heavy
TF	504.760032	546.301338	TF.YLGEEYVK.+2y4.heavy
TF	368.205421	678.382102	TF.GDVAFVK.+2y6.light
TF	368.205421	563.355159	TF.GDVAFVK.+2y5.light
TF	372.212521	686.396301	TF.GDVAFVK.+2y6.heavy
TF	372.212521	571.369358	TF.GDVAFVK.+2y5.heavy

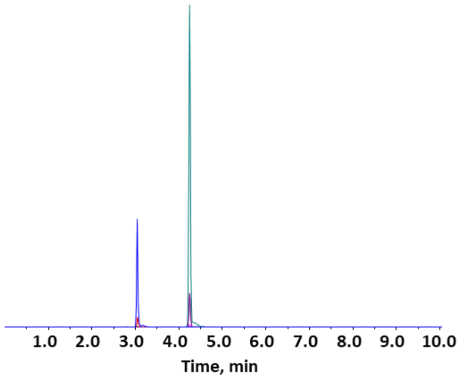

Figure S7. Representative chromatograms for targeted proteomics. (a) Representative total chromatogram of the biological sample in targeted proteomics method. (b) Peptide ion chromatograms and transition lists are used for protein identification and quantification.
